# Deficiency of NPGPx, an oxidative stress sensor, leads to obesity in mice and human

**DOI:** 10.1002/emmm.201302679

**Published:** 2013-07-04

**Authors:** Yi-Cheng Chang, Yu-Hsiang Yu, Jin-Yuh Shew, Wei-Jei Lee, Juey-Jen Hwang, Yen-Hui Chen, Yet-Ran Chen, Pei-Chi Wei, Lee-Ming Chuang, Wen-Hwa Lee

**Affiliations:** 1 Genomics Research Center, Academia SinicaTaipei, Taiwan; 2 Graduate Program of Translational Medicine, National Taiwan UniversityTaipei, Taiwan; 3 Department of Internal Medicine, National Taiwan University HospitalTaipei, Taiwan; 4 Department of Surgery, Min-Sheng General HospitalTaoyan, Taiwan; 5 Institute of Biomedical Sciences, Academia SinicaTaipei, Taiwan; 6 Agricultural Biotechnology Research Center, Academia SinicaTaipei, Taiwan; 7 Department of Biological Chemistry, University of CaliforniaIrvine, CA, USA

**Keywords:** adipogenesis, C/EBPβ, *N*-acetylcysteine, NPGPx, oxidative stress

## Abstract

Elevated oxidative stress is closely associated with obesity. Emerging evidence shows that instead of being a consequence of obesity, oxidative stress may also contribute to fat formation. Nonselenocysteine-containing phospholipid hydroperoxide glutathione peroxidase (NPGPx) is a conserved oxidative stress sensor/transducer and deficiency of NPGPx causes accumulation of reactive oxygen species (ROS). In this communication, we show that NPGPx was highly expressed in preadipocytes of adipose tissue. Deficiency of NPGPx promoted preadipocytes to differentiate to adipocytes via ROS-dependent dimerization of protein kinase A regulatory subunits and activation of CCAAT/enhancer-binding protein beta (C/EBPβ). This enhanced adipogenesis was alleviated by antioxidant *N*-acetylcysteine (NAC). Consistently, NPGPx-deficient mice exhibited markedly increased fat mass and adipocyte hypertrophy, while treatment with NAC ablated these phenotypes. Furthermore, single nucleotide polymorphisms (SNPs) in human *NPGPx* gene, which correlated with lower NPGPx expression level in adipose tissue, were associated with higher body mass index (BMI) in several independent human populations. These results indicate that NPGPx protects against fat accumulation in mice and human via modulating ROS, and highlight the importance of targeting redox homeostasis in obesity management.

Deficiency of the glutathione peroxidase NPGPx increases ROS levels in preadipocytes and promotes adipocyte differentiation via increasing oxidative stress and consequent increased fat mass and adipocyte hypertrophy.

## INTRODUCTION

Obesity is a global epidemic with huge impact on human health. Epidemiological studies have consistently observed a close association between oxidative stress and excessive fat accumulation (Festa et al, 2001; Keaney et al, 2003; Urakawa et al, 2003). Although the causal relationship underlying this association remained uncertain, fat accumulation with subsequent inflammatory response was generally considered as the sources of oxidative stress (Furukawa et al, 2004; Weisberg et al, 2003). However, emerging evidence suggests that instead of being a consequence, oxidative stress may be a prerequisite for adipogenesis. Reactive oxygen species (ROS) level is increased during adipogenesis (Tormos et al, 2011). Administration of mitochondria-targeted antioxidants reduces adipocyte differentiation while exogenous H_2_O_2_ promotes adipocyte differentiation in mesenchymal stem cells (Tormos et al, 2011). Nevertheless, the physiological relevance of these observations have not been tested *in vivo* and the how ROS is regulated during normal adipocyte differentiation is little known.

Several physiological processes including mitochondrial oxidative phosphorylation and protein folding at the endoplasmic reticulum (ER) generate superoxide (O^-^_2_). Superoxide is first converted to H_2_O_2_ by superoxide dismutase and then to water by antioxidant enzymes such as catalase, thioredoxin peroxidase, and glutathione peroxidase (GPx) (D'Autreaux & Toledano, 2007). Among them, the GPx represent an important protein family discovered in nearly all kingdoms of life. To date, eight homologous GPx members (GPx1–8) have been identified in mammals (Toppo et al, 2008). GPx1 is ubiquitously distributed. However, GPx1 knockout mice develop normally without evidence of increased oxidative stress (Ho et al, 1997). GPx2 (gastrointestinal GPx) is expressed mainly in the gastrointestinal epithelium (Chu et al, 1993). GPx3 (plasma GPx) is an extracellular enzyme secreted from the kidney into the circulating blood (Yoshimura et al, 1991). GPx5 is a selenocysteine-independent GPx expressed specifically in the epididymis (Chabory et al, 2009) and GPx6 is found almost exclusively in the olfactory epithelium. In contrast to GPx1, 2, 3, 5 and 6 which appeared relatively recently in evolution, phylogenic analysis revealed that GPx4, 7 and 8 are ancient with conserved sequences in protozoa and invertebrate (Toppo et al, 2008). GPx4 (phospholipid hydroperoxide GPx) is a membrane-bound protein that accepts phospholipid hydroperoxides in membranes as substrates with important function in spermatogenesis (Imai et al, 2009). GPx7, also known as nonselenocysteine-containing phospholipid hydroperoxide glutathione peroxidase (NPGPx, Gene ID: 67305), and GPx8 share a similar structure but without glutathione-binding domain and have no apparent enzymatic activity (Nguyen et al, 2011). NPGPx was identified as an oxidative stress sensor/transducer that senses and transmits cellular ROS to downstream mediators to reduce ROS accumulation (Nguyen et al, 2011; Peng et al, 2012; Utomo et al, 2004; Wei et al, 2012), while the biological function of GPx8 remains to be shown. In contrast to GPx1 knockout mice, which have little obvious phenotype, loss of NPGPx increases oxidative stress in mice (Wei et al, 2012), suggesting NPGPx is essential for maintaining redox homeostasis.

In this communication, we show that NPGPx was highly expressed in preadipocytes but not in mature adipocytes. Deficiency of NPGPx facilitated preadipocytes to differentiate to adipocytes through ROS-dependent activation of PKA/C/EBPβ signalling pathway. Consistently, NPGPx-deficient mice exhibited increased white fat mass and adipocyte hypertrophy that can be prevented by antioxidant *N*-acetylcysteine (NAC). Furthermore, single nucleotide genetic polymorphisms near the human *NPGPx* gene, which correlated with lower NPGPx expression in white adipose tissue, were associated with increased adiposity in several independent human populations. These results indicate that NPGPx plays a critical role in adiposity mediated through redox homeostasis.

## RESULTS

### NPGPx deficiency mediates adipogenesis through ROS

**Figure 1:**
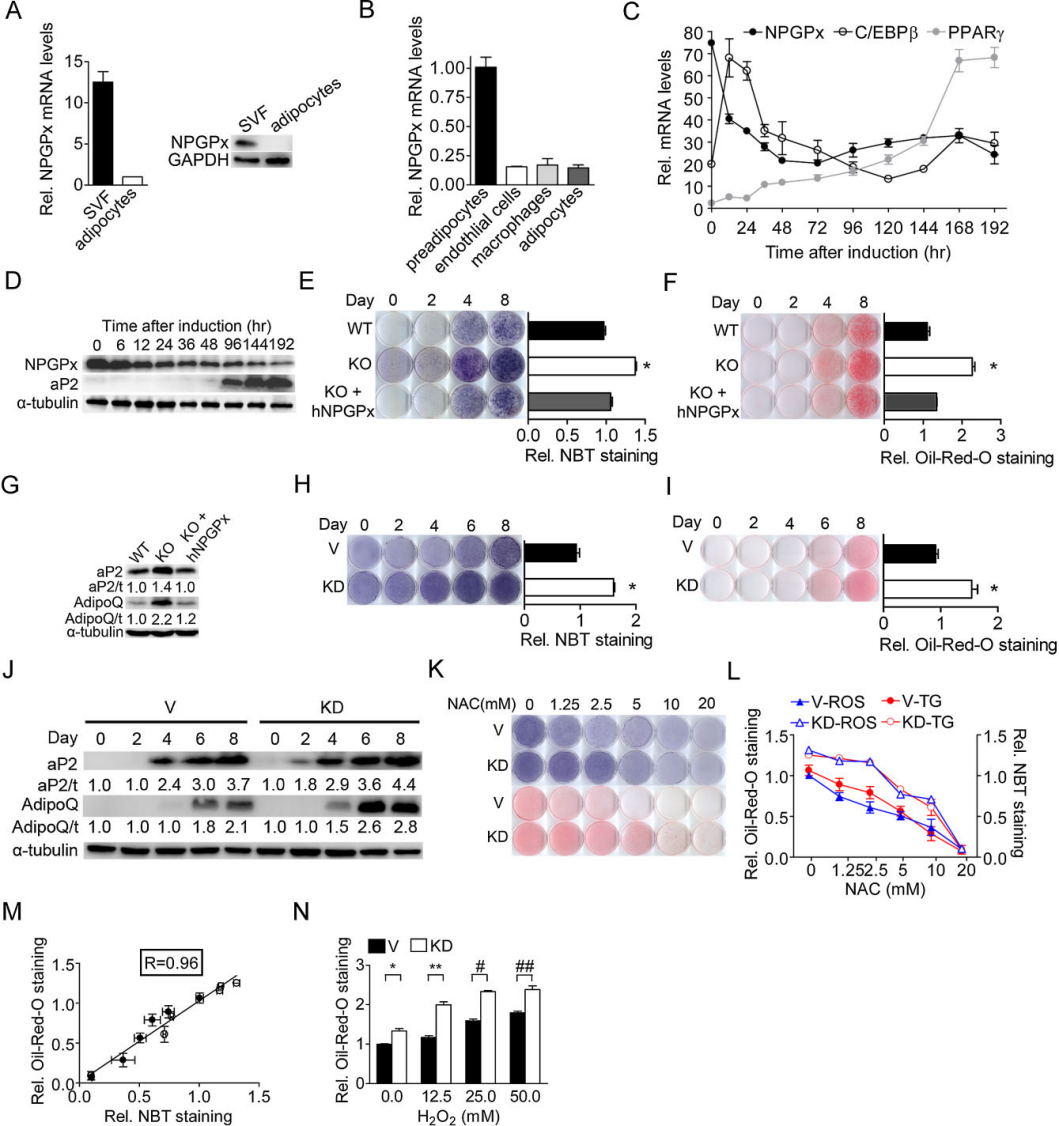
**NPGPx is a suppressor of adipogenesis via ROS regulation**
Relative NPGPx expression assayed by qRT-PCR (left panel) and Western blots (right panel) in the stromal vascular fraction (SVF) and adipocyte fraction isolated from the gonadal fat of C57BL/6 mice (*n* = 3 per group).Relative NPGPx expression in preadipocytes (Lin^−^CD34^+^CD29^+^Sca1^+^), endothelial cells (CD31^+^), macrophages (CD11b^+^) and adipocytes of adipose tissue measured by in C57BL/6 mice (*n* = 3 per group).*NPGPx*, CCAAT/enhancer-binding protein β (C/EBPβ) and peroxisome proliferator-activated receptor γ (PPARγ) mRNA level during induced 3T3-L1 adipogenesis (*n* = 5 per group).NPGPx expression assayed by Western blots during 3T3L1 adipogenesis.NBT reduction staining of wild-type SVF cells (WT), NPGPx-knockout SVF cells (KO) and NPGPx-knockout SVF cells expressing human NPGPx (KO + hNPGPx) (left panels). Quantification of NBT reduction stains 8 days after induction (*n* = 3 per group) (right panels). *p = 0.0002 for WT vs. KO by independent Student's *t*-test.Oil Red O stain of WT, KO and KO + hNPGPx SVF (left panels). Quantification of Oil Red O stain 8 days after induction (*n* = 3 per group) (right panels). *p = 0.0002 for WT vs. KO by independent Student's *t*-test.Western blots showing *ap2* and *adiponectin* expression in WT, KO and KO + hNPGPx SVF cells 8 days after induction.NBT reduction stain of NPGPx knockdown (KD) and control (V) 3T3-L1 preadipocytes after adipogenic induction (left panels). Quantification of NBT reduction stain at day 8 (*n* = 3 per group) (right panels). *p = 0.0004 by independent Student's *t*-test.Oil Red O staining of KD and V 3T3-L1 preadipocytes after adipogenic induction (left panels). Quantification of Oil Red O stain at day 8 (*n* = 3 per group) (right panels). *p = 0.0005 by independent Student's *t*-test.Western blots showing *ap2* and *adiponectin* expression in NPGPx KD and V 3T3-L1 preadipocytes after adipogenic stimulation.NBT reduction stain and Oil Red O stain of NPGPx KD and V 3T3-L1 cells 8 days after induction. Cells were treated at with NAC at various doses the first 2 days after induction.Quantification of NBT reduction stain and Oil Red O stain in (K) (*n* = 3 per group).Correlation (*R* = 0.96) between ROS levels (Rel. NBT staining) and triglyceride contents (Rel. Oil-Red-O staining) in NPGPx KD and V 3T3-L1 cells 8 days after induction.Quantification of Oil Red O staining of NPGPx KD and V 3T3-L1 cells 8 days after induction. Cells were treated with H_2_O_2_ at different concentrations the first 2 days after induction (*n* = 3 per group). *p = 0.006, **p = 0.006, #*p* < 0.0001, ##*p* < 0.0001 by independent Student's *t*-test. All values are presented as means ± S.E.M.

To investigate the physiological role of NPGPx, we first compared the tissue expression patterns of GPx gene family. Among all GPx members, NPGPx was highly enriched in white adipose tissue (Supporting Information Fig S1A–I). Within white adipose tissue, NPGPx was mainly expressed in the stromal vascular fraction (SVF), specifically in preadipocytes, but little in mature adipocytes, macrophages or endothelial cells (Fig 1A, B). Expression of NPGPx in 3T3-L1 preadipocytes declined rapidly within the first 24 h during the course of induced adipocyte differentiation, followed by the rise of C/EBPβ and peroxisome proliferator-activated receptor gamma (PPARγ) (Fig 1C, D). These expression profiles implicated that NPGPx may have a role in adipogenesis.

To test this possibility, SVF cells isolated from white adipose tissue of NPGPx knockout or wild-type mice were induced for adipocyte differentiation by hormonal stimulation. Throughout the differentiation course, SVF cells from NPGPx knockout mice showed consistently higher ROS levels, as measured by nitroblue tetrazolium (NBT) reduction staining (Fig 1E) and were more prone to differentiate into adipocytes (Fig 1F) than that of wild-type mice with higher expression of adipogenic marker genes including *ap2* and *adiponectin* (Fig 1G). Ectopic re-expression of human *NPGPx* gene in SVF cells from knockout mice suppressed ROS levels (Fig 1E) and reduced differentiation ability (Fig 1F, G) (Supporting Information Fig S2A). Similarly, knockdown of NPGPx in 3T3-L1 preadipocytes increased ROS levels (Fig 1H) and enhanced adipogenesis (Fig 1I) (Supporting Information Fig S2B), while ectopic overexpression of NPGPx in 3T3-L1 cells reduced ROS levels (Supporting Information Fig S3A, B) and suppressed adipogenesis (Supporting Information Fig S3C, D). Consistently, both primary SVF cells and adipocytes isolated from white adipose tissue of NPGPx knockout mice displayed significantly higher ROS levels than those from wild-type littermates (Supporting Information Fig S4A). These findings indicate that NPGPx deficiency elevates ROS level and enhances adipocyte differentiation.

To test whether the pro-adipogenic effect resulting from NPGPx deficiency is mediated by the increased ROS, we thought to use antioxidants or pro-oxidants to assay the effect on adipogenesis. The ROS level and differentiation ability were higher in NPGPx-knockdown 3T3-L1 cells than in control cells in response to different dose of NAC treatment (Fig 1K, L). Quantitative measurements indicated that the ROS levels correlated strongly (*r* = 0.96, *p* < 0.0001) with the degree of adipocyte differentiation for various doses of NAC (Fig 1 M). Similar suppressive effects were observed using tiron, a superoxide scavenger (Han & Park, 2009) and 4-phenylbutyrate (PBA), a chemical chaperone that reduces ROS levels (Liu et al, 2002) (Supporting Information Fig S4B, C). In contrast, adding different dose of H_2_O_2_ during the differentiation course, the NPGPx-knockdown 3T3-L1 cells appeared to be more differentiated than the control cells (Fig 1N). These data indicate that NPGPx participates in adipogenesis through modulating cellular ROS level.

### High level of ROS enhances adipogenesis by activating C/EBPβ

**Figure 2:**
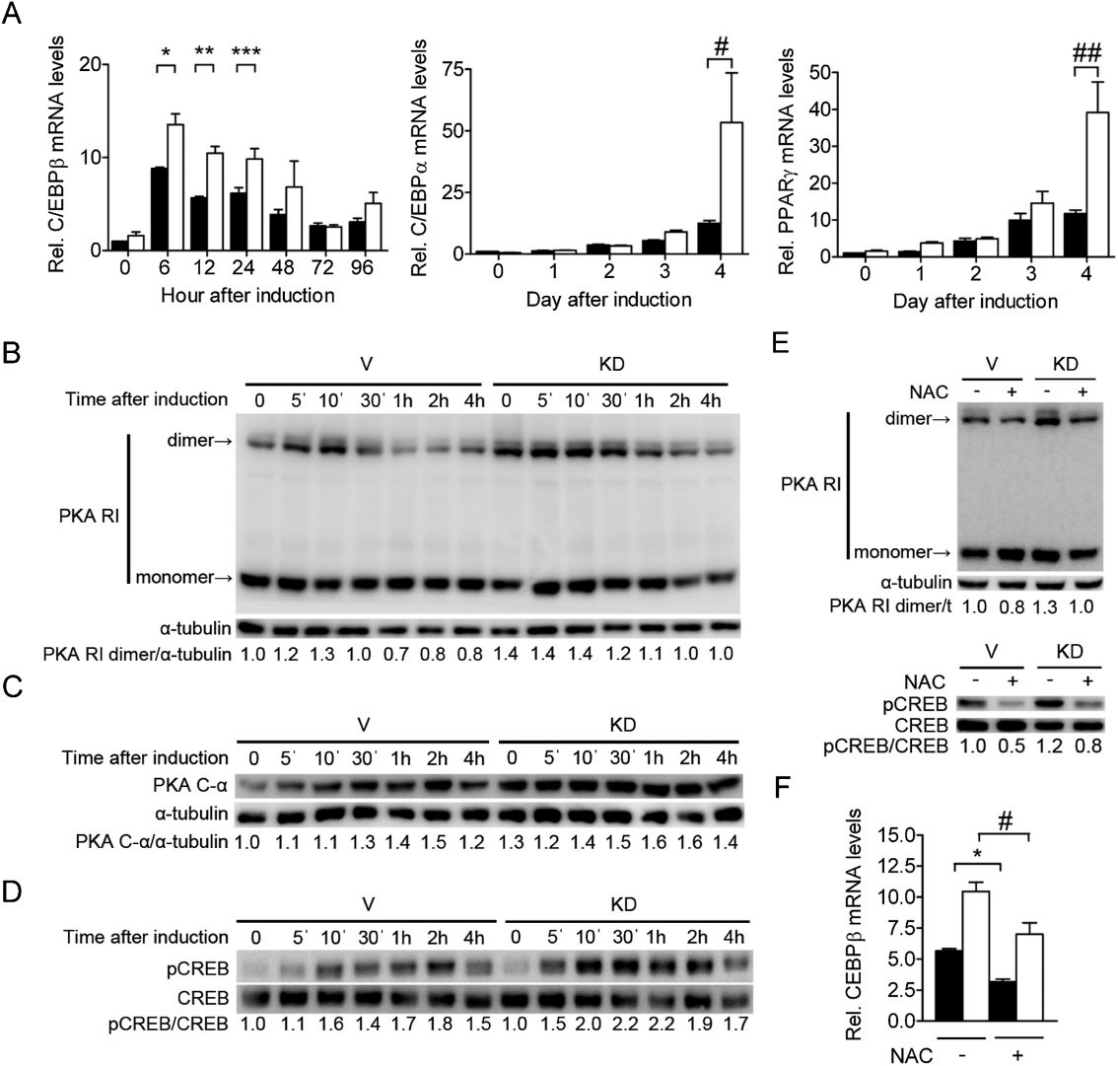
**NPGPx deficiency promotes adipogenesis via ROS-dependent dimerization of protein kinas A regulatory subunits and transcription of C/EBP β**
Expression of C/EBP β, C/EBP α and PPAR γ measured by qRT-PCR in NPGPx knockdown (KD) and control (V) 3T3-L1 preadipocytes after adipogenic stimulation (*n* = 5 per group). *p = 0.03, **p = 0.007, ***p = 0.03, #*p* = 0.04, ##*p* = 0.008 by independent Student's *t*-tests.Western blots of the regulatory subunits of protein kinase A (PKA RI) (B) (non-reducing gel), the monomeric protein kinase A catalytic subunits (PKA C-α) (reducing gel) (C), and phosphorylated CREB (reducing gel) (D) in NPGPx KD and V 3T3-L1 cells after adipogenic stimulation.Western blots showing the dimer/monomer of PKA regulatory subunits (non-reducing gel, upper panel) and phosphorylated CREB/CREB (reducing gel, lower panel) in NPGPx KD and V 3T3-L1 preadipocytes 30 min after induction with or without *N*-acetylcysteine (NAC) 10 mM.C/EBP β expression measured by qRT-PCR in NPGPx KD and V 3T3-L1 preadipocytes 12 h after induction with or without NAC 10 mM (*n* = 5 per group). *p = 0.002 and #*p* = 0.02 by independent Student's *t*-tests. Open bars (□) denote NPGPx knockdown 3T3-L1 cells while filled bars (▪) denote control cells. All values are presented as means ± S.E.M. [Correction added after publication on 4 July 2013: The phrase “by dependent Student's *t*-tests” in the legend of Fig. 2F has been corrected to “by independent Student's *t-*tests”].

To explore the molecular mechanism by which ROS enhances adipogenesis, we assessed the expression of key transcriptional factors involved in adipocyte differentiation (Yeh et al, 1995). C/EBPβmRNA expression was significantly increased in NPGPx-knockdown 3T3-L1 preadipocytes after adipogenic induction (Fig 2A). Expression of downstream transcriptional factors including C/EBPα and PPARγ mRNA was also higher in NPGPx-knockdown cells (Fig 2A). These results indicate NPGPx deficiency in 3T3-L1 preadipocytes increases the expression of key transcription factors involved in adipogenesis.

C/EBPβ expression is primarily regulated through the PKA/CREB signalling pathway (Rosen & MacDougald, 2006). PKA is a hetero-tetramer comprising of 2 regulatory subunits harbouring potential ROS-sensitive cysteinyl residues and 2 catalytic subunits (Brennan et al, 2004; Weerapana et al, 2010). After adipogenic hormonal stimulation, cAMP binds to the PKA regulatory subunit, causing the breakdown of the PKA complex and the release of the PKA catalytic subunit. The PKA catalytic subunits phosphorylate cyclic AMP response element-binding protein (CREB), which in turn binds to the promoter of C/EBP β. Therefore, we tested whether NPGPx depletion affect the key steps of this cascade. We found that NPGPx deficiency promoted the dimerization of PKA regulatory subunits (Fig 2B), the release of the monomeric PKA catalytic subunits (Fig 2C) and the CREB phosphorylation (Fig 2D). Antioxidant NAC treatment suppressed the dimerization of PKA regulatory subunits and the CREB phosphorylation (Fig 2E), leading to the reduction of C/EBP β expression (Fig 2F). These data indicate that NPGPx deficiency increases C/EBP β expression through PKA signalling pathway in an ROS-dependent manner.

**Figure 3:**
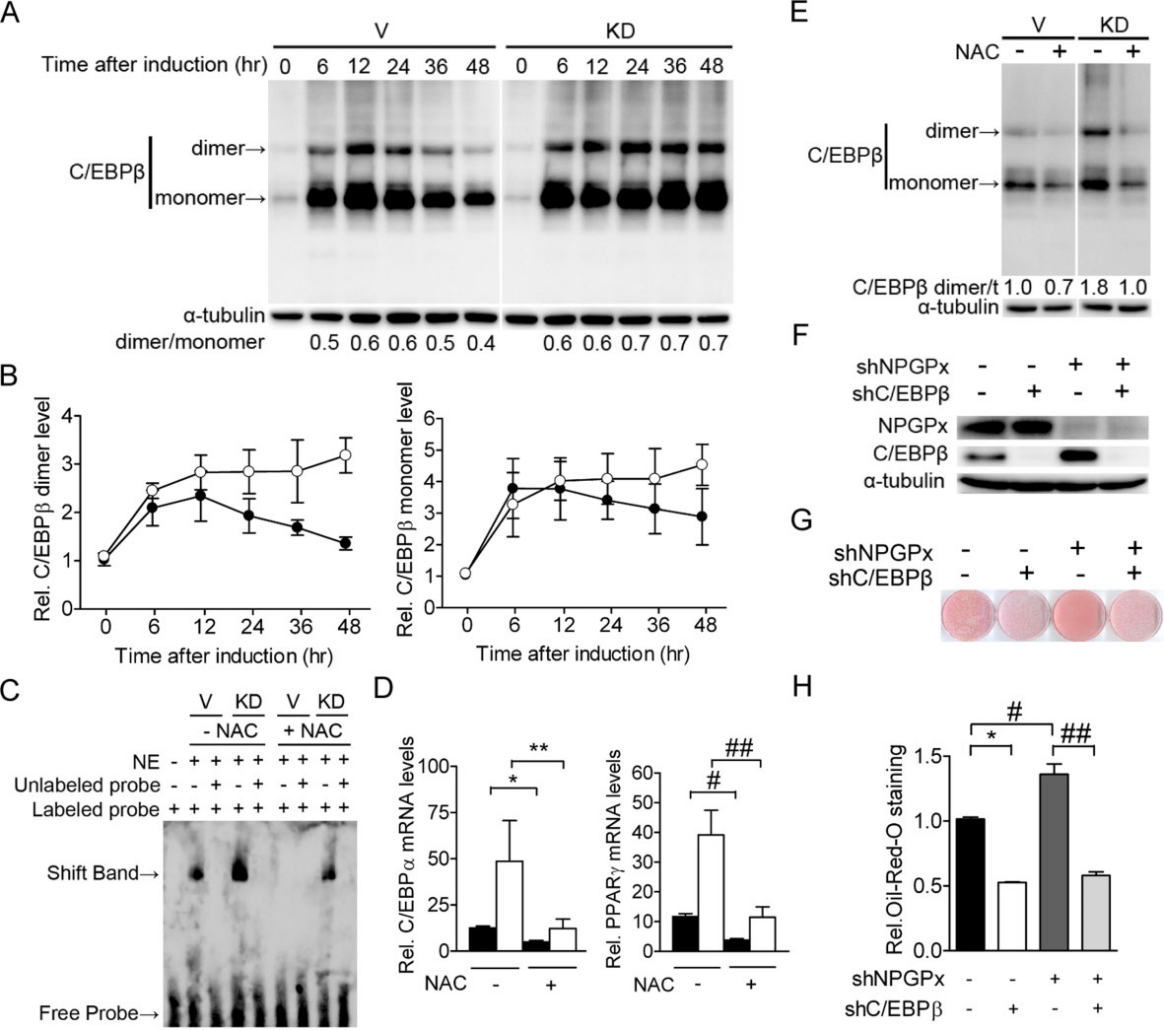
**NPGPx deficiency enhances dimerization of C/EBP β and its transcriptional activity**
Western blots of C/EBP β dimer/monomer (non-reducing gel) in NPGPx knockdown (KD) and control (V) 3T3-L1 preadipocytes after adipogenic induction.Quantification of the C/EBP β dimer (left panel) and monomer (right panel) in (A) (*n* = 3 per group).Electrophoretic mobility shift assay (EMSA) with nuclear extract from NPGPx KD and V 3T3-L1 preadipocytes 48 h after induction with or without NAC. The extracts were reacted with biotinylated oligonucleotides containing the C/EBP β-binding sequence of the C/EBP α promoter and were separated by native polyacrylamide electrophoresis gel native. [Correction added after publication on 4 July 2013: The indication in the Figure (Fig. 3C) that labeled probe was not included in the first lane (shown by the minus symbol (−) for labeled probe above the first lane) has been corrected by replacement with the plus symbol (+)].C/EBPα and PPARγ expression measured by qRT-PCR in NPGPx KD and V 3T3-L1 preadipocytes 4 days after induction with or without treatment with NAC 10 mM. *p = 0.01, **p = 0.03, #*p* = 0.002 and ##*p* = 0.03 by independent Student's *t*-tests.Western blots (non-reducing gel) showing the C/EBP β dimer and monomer in NPGPx KD and V 3T3-L1 preadipocytes 48 hrs after induction with or without NAC 10 mM.Western blot showing NPGPx and C/EBP β expression in 3T3-L1 preadipocytes with NPGPx-knockdown (shNPGPx) and/or C/EBP β-knockdown (shC/EBP β)Oil Red O stain of 3T3-L1 preadipocytes with NPGPx-knockdown and/or C/EBP β-knockdown 8 days after induction.Quantification of (G) (*n* = 3 per group). *p < 0.0001, #*p* = 0.01 and ##*p* = 0.0008 by Student's *t*-tests. Open circles (○) or open bars (□) denote NPGPx knockdown 3T3-L1 cells. Filled circles (●) or filled bars (▪) denote control cells. All values are presented as means ± S.E.M.

It has been reported that C/EBPβ is activated by ROS to form a dimer through the formation of intermolecular disulphide bonds. This conformational change increases its DNA binding activity (Tang & Lane, 2012). We then tested whether NPGPx deficiency has any effect on C/EBP β dimerization and its DNA binding activity. As shown in Fig 3A, B, the expression of C/EBPβ monomer and dimer peaked at 12 h after adipogenic induction and declined afterwards in control cells. In contrast, the expression of C/EBPβ monomer and dimer persisted beyond 24 h in NPGPx-deficient cells. In addition, the dimer to monomer ratio of C/EBPβ was also increased in NPGPx-deficient cells (Fig 3A). The increased dimerization of C/EBPβ was associated with increased DNA binding to the promoter of C/EBPα (Fig 3C) and increased expression of downstream C/EBPα and PPARγ (Fig 3D). Treatment with NAC reduced the dimerization (Fig 3E) and DNA binding activity of C/EBP β (Fig 3C), and suppressed the expression of C/EBPα and PPARγ (Fig 3D).

In addition to transcriptional activation of the adipogenic cascade, C/EBPβ is also required for mitotic clonal expansion in early adipogenesis (Tang et al, 2003). We then tested this possibility by measuring cell proliferation in early adipogenesis and found an increase of proliferation in NPGPx-knockdown cells after induction, which was suppressed by NAC (Supporting Information Fig S5). The increased mitosis in early adipocyte differentiation explains the increased cellular proliferation found in NPGPx knockout mice.

To affirm that C/EBPβ is responsible for the pro-adipogenic effect resulting from NPGPx deficiency, we compared the effect of NPGPx on adipogenesis in the presence or absence of C/EBP β. We found that the pro-adipogenic effect conferred by NPGPx deficiency was abolished when C/EBPβ was absent (Fig 3F–H), indicating that C/EBPβ is essential for mediating the pro-adipogenic effect resulting from NPGPx deficiency. Taken together, these data suggested that NPGPx deficiency increases both the expression and dimerization of C/EBP β through a ROS-mediated mechanism, thereby enhancing adipocyte differentiation.

### NPGPx-deficient mice exhibit increased mass of white adipose tissue and adipocyte hypertrophy

**Figure 4:**
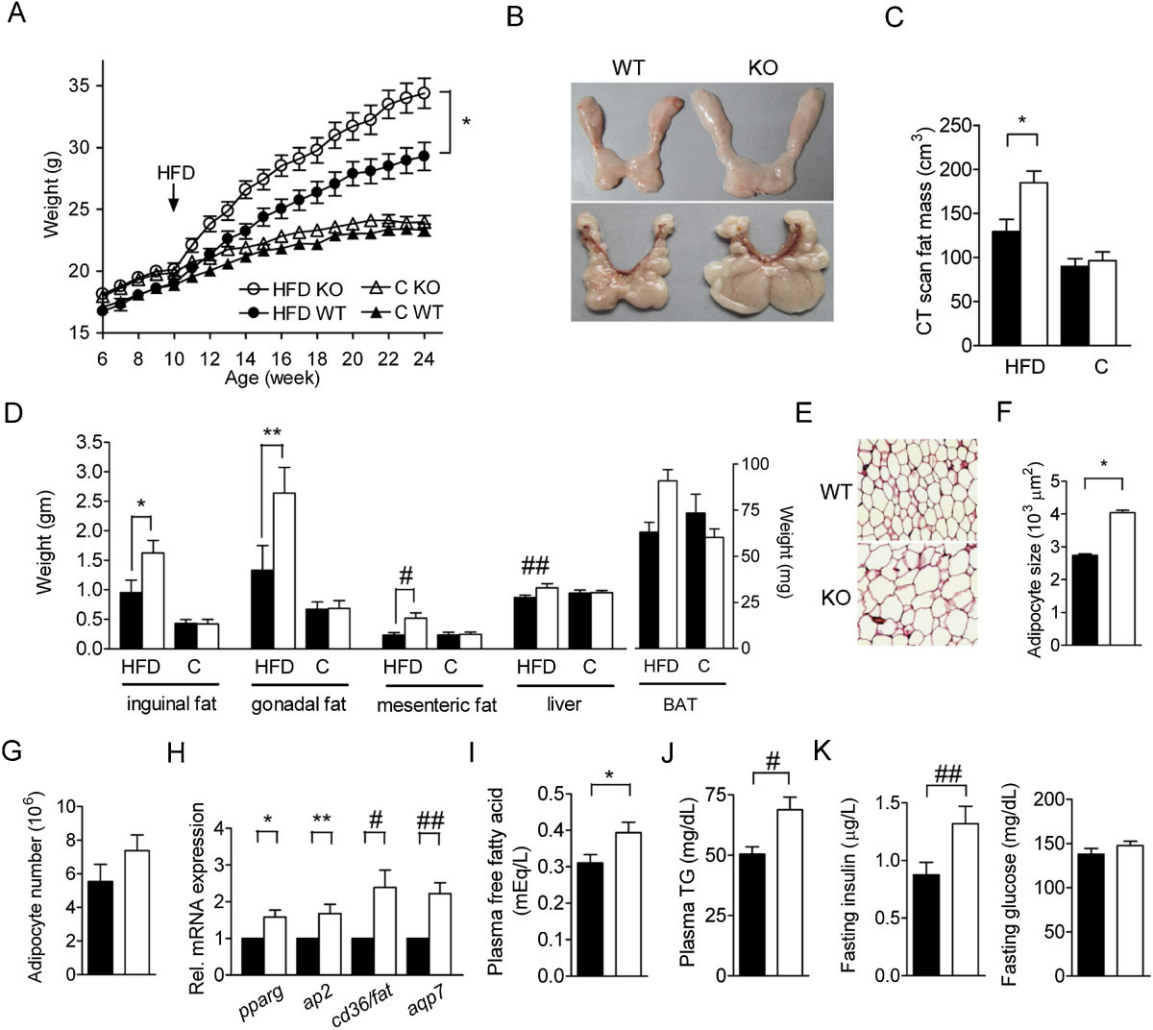
**Deficiency of NPGPx enhances adipogenesis *in vivo***Body weight of NPGPx knockout (KO) mice and wild-type littermates (WT) fed on standard chow diet (C) or high-fat diet (HFD) (*n* = 27–30 per group). The HFD was started at the age of 10 weeks. *p = 0.017 by repeated measures ANOVA.Representative photograph inguinal fat pads (upper panel) and gonadal fat pads (lower panel) of 24-week-old NPGPx KO or NPGPx WT mice fed on HFD.Fat mass of 24 week-old mice measured by micro-CT (*n* = 10 per group). *p = 0.009 by independent Student's *t*-test.Weight of white fat pads (inguinal fat, gonadal fat and mesenteric fat), brown fat pads (BAT) and the liver (*n* = 10 per group). *p = 0.03, **p = 0.04, #*p* = 0.009 and ##p = 0.06 by independent Student's *t*-tests.Representative haematoxylin and eosin (H&E) staining of the gonadal fat tissue of WT or KO mice fed on HFD.Adipocyte size and (F) estimated adipocyte number (G) of the gonadal fat pads of 24 week-old mice fed on HFD (*n* = 15 per group). *p < 0.0001 by independent Student's *t*-test.Relative expression of adipogenesis marker genes (*pparg*, *ap2*, *cd36/fat* and *aqp7*) measured by qRT-PCR in the gonadal fat pads of 24-week-old mice fed on HFD (*n* = 5 per group). *p = 0.01, **p = 0.02, #*p* = 0.01, ##*p* = 0.004 by paired Student's *t*-test.Fasting plasma free fatty acid (I), fasting triglycerides (TG) (J), fasting insulin and glucose levels (K) in 24-week-old mice fed on HFD. *p = 0.02, #*p* = 0.003 and ##*p* = 0.03 by independent Student's *t*-tests. Open circles (○), triangles (△) or open bars (□) denote knockout mice. Filled circles (●), filled triangles (▴) or filled bars (▪) denote wild-type mice. All values are presented as means ± S.E.M.

Next, we sought to determine whether NPGPx affects adipogenesis *in vivo*. On standard chow diet containing 13% calories from fat, the body weight of NPGPx knockout mice was not different from those of wild-type littermates (Fig 4A). However, when mice were fed the high-fat diet (HFD) containing 60% calorie from fat, the mutant mice became significantly heavier than wild-type littermates (Fig 4A, B). Micro-computed tomography (CT) revealed higher body fat percentages of NPGPx knockout mice compared to wild-type littermates on HFD (Fig 4C). White fat pads, including inguinal, gonadal and mesenteric fat, were substantially increased (∼2-fold) in knockout mice (Fig 4D). Histological examination revealed that the adipocyte size (adipocyte hypertrophy) (Fig 4E, F) and, to a lesser extent, the adipocyte number (adipocyte hyperplasia) was increased in the gonadal fat of knockout mice on an HFD (Fig 4G), accompanied with an increased expression of adipogenic marker genes such as *pparg*, *ap2*, *cd36/fat* and *aqp7* (Fig 4H). Increased cell proliferation measured by bromodeoxyuridine (BrdU) uptake was observed in the gonadal fat tissue of knockout mice on HFD (Supporting Information Fig S6). These data indicate that NPGPx deficiency facilitated cell proliferation and hyperplasia in fat tissue of mice. The increased adiposity was associated with elevated fasting plasma free fatty acids (Fig 4I), triglycerides (Fig 4J) and insulin levels (Fig 4K). Fasting glucose was not changed (Fig 4K). Increased macrophage infiltration with higher expression of inflammatory marker genes (*tnfα*, *il6* and *il1β*) was also observed in fat tissue of these mice (Supporting Information Fig S7).

No difference in food intake (Supporting Information Fig S8A) between knockout and wild-type mice was noted. Reduced energy expenditure was observed in knockout mice during the dark/active phase as compared to wild-type mice on HFD, whereas no difference was observed on chow diet (Supporting Information Fig S8B). Spontaneous ambulatory activity was also decreased in knockout mice on HFD but not in chow diet (Supporting Information S9A). However, exercise capability assayed by exercise endurance test or rotarod test was not impaired in these mice (Supporting Information Fig S9B, C). We cannot detect defect of fatty acid beta-oxidation (Supporting Information Fig S9D) and mitochondrial oxidative phosphorylation (Supporting Information Fig S9E) in primary myoblasts or fibroblasts isolated from NPGPx knockout mice. Furthermore, rectal temperature and expression of genes involved in thermogenesis in brown adipose tissue (BAT) were not altered in knockout mice (Supporting Information Fig S9F, G).

### Antioxidant NAC suppresses adipogenesis in NPGPx-deficient mice

**Figure 5:**
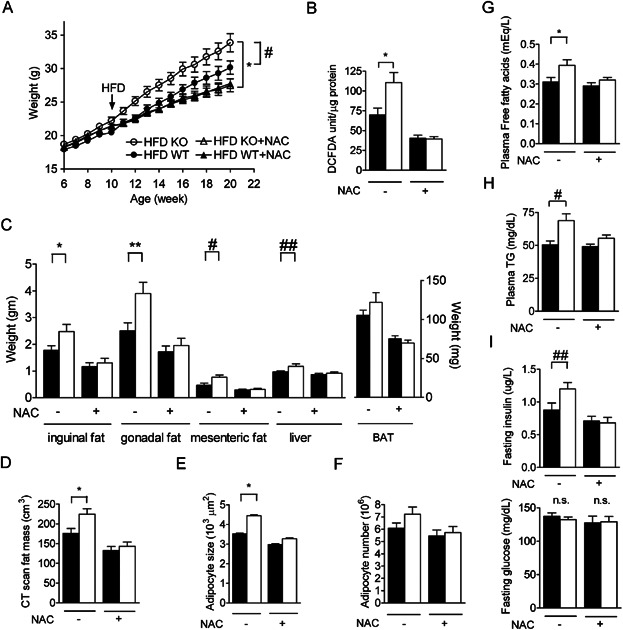
***N*-acetylcysteine (NAC) suppresses fat formation of NPGPx-deficient mice**Body weight of NPGPx knockout (KO) mice and wild-type (WT) littermates on high-fat diet (HFD) with or without NAC treatment (*n* = 24–30 per group). HFD and the NAC treatment were started at the age of 10 weeks. *p = 0.003 and #*p* = 0.04 by repeated measures ANOVA.ROS levels in the gonadal fat pads of 24-week-old mice measured by DCFDA fluorescence (*n* = 5 per group). *p = 0.01 by independent Student's *t*-test.Weight of white fat pads (inguinal fat, gonadal fat and mesenteric fat), brown fat pads (BAT), and the liver from 24-week-old mice (*n* = 15 per group). *p = 0.04, **p = 0.01, #*p* = 0.02, ##*p* = 0.04 by independent Student's *t*-tests.Fat mass of 24-week-old mice measured by microCT (*n* = 15 per group). *p = 0.02 by independent Student's *t*-test.Adipocyte size (E) and estimated adipocyte number (F) in the gonadal fat pad of 24-week-old mice (*n* = 15 per group). *p < 0.0001 by independent Student's *t*-test.Fasting plasma free fatty acid (G), fasting plasma TG (triglycerides) (H), fasting insulin and glucose levels (I) of 24-week-old mice (*n* = 15 per group). *p = 0.02, #*p* = 0.003 and ##*p* = 0.03 by independent Student's *t*-tests. Open circles (○), triangles (△) or open bars (□) denote knockout mice. Filled circles (●), filled triangles (▴) or filled bars (▪) denote wild-type mice. All values are presented as means ± S.E.M.

To test whether anti-oxidant NAC can reduce adiposity in NPGPx-deficient mice, mice were given either regular drinking water or water supplemented with NAC. NAC treatment suppressed fat accumulation and reduced ROS levels in both mutant and wild-type mice on HFD (Fig 5A–C). The differences in ROS levels and fat accumulation between mutant and wild-type mice were completely mitigated by the NAC treatment (Fig 5A–C), as were differences in body fat percentage, fat pad weights and adipocyte size (Fig 5D–F). Similar effects were also found for fasting plasma free fatty acids (Fig 5G), triglycerides (Fig 5H) and insulin (Fig 5I) levels. Fasting glucose level was not changed (Fig 5I). NAC treatment did not alter food intake (Supporting Information Fig S10A) or energy expenditure in either mutant or wild-type mice (Supporting Information Fig S10B).

### Association of *NPGPx* genetic variants with body mass index (BMI) in humans

**Figure 6:**
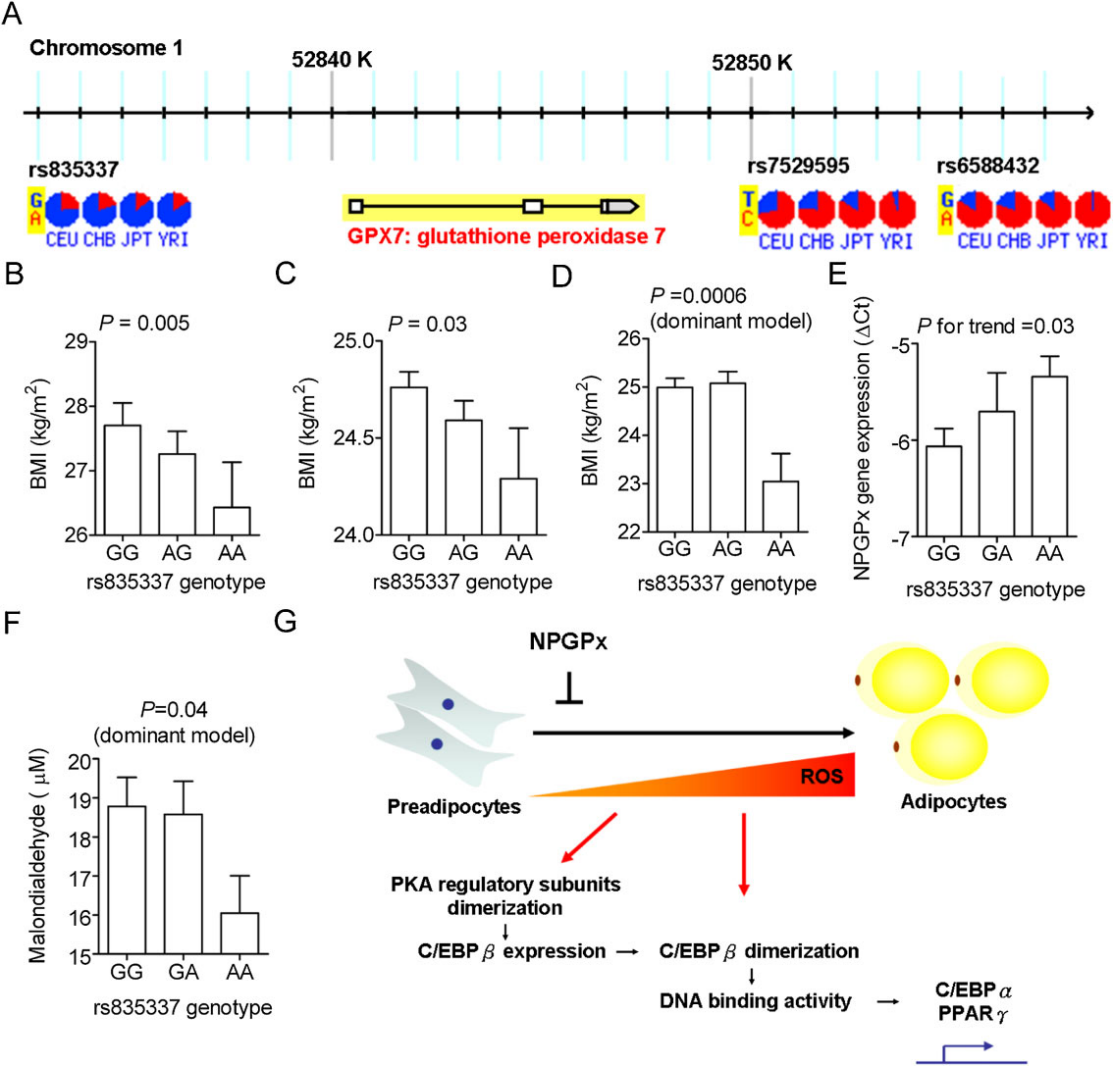
**Association of *NPGPx* genetic polymorphisms with body mass index (BMI) in human**Genomic location of single nucleotide polymorphism (SNP) adjacent to the human *NPGPx* gene. The allele frequency of each SNP in the HapMap database is shown in pie charts. YRI: Yoruba in Ibadan, Nigeria; JPT: Japanese in Tokyo, Japan; CHB: Han Chinese in Beijing, China; CEU: CEPH Utah residents with ancestry from northern and western Europe.The association of SNP rs835337 with BMI in 1430 adults of the British 1958 Birth Cohort (B), 4758 adults in the North Finland Birth Cohort (C) and 760 Han Chinese (D).Association of SNP rs835337 genotypes with NPGPx expression measured by qRT-PCR in abdominal adipose tissue of 84 Han Chinese.Association of SNP rs835337 genotypes with serum malondialdehyde levels.Proposed mechanism by which NPGPx modulates adipogenesis. All values are presented as means ± S.E.M.

To test whether *NPGPx* also plays a role in human adiposity, we analysed the association of single nucleotide polymorphisms (SNPs) near the human *NPGPx* gene with adiposity in independent human populations. The genetic variant rs835337, which was located upstream the human *NPGPx* gene (Fig 6A), was significantly associated with the BMI in 1424 adults of the British 1958 Birth Cohort (Fig 6C, *p* = 0.005, Supporting Information Table S1) and 4758 adults in the North Finland Birth Cohort (Fig 6B, *p* = 0.03, Supporting Information Table S1). This variant was also associated with BMI in 760 Han Chinese adults (Fig 6D, *p* = 0.006 in dominant genetic model, corrected *p* = 0.007 for multiple genetic models, Supporting Information Table S1). Meta-analysis pooling these three independent studies showed a significant association between rs835337 and BMI (*p* = 0.0004) (Supporting Information Fig S11A). This SNP is located within a large linkage disequilibrium (LD) block spanning the entire *NPGPx* gene. Other SNPs in strong LD with rs835337, including rs7529595 and rs6588432 downstream of the *NPGPx* gene, were also associated with BMI (*p* = 0.002 and 0.0003, respectively) (Supporting Information Fig S11B, C and Table S1). Importantly, the risk-conferring G allele of rs835337 was associated with lower *NPGPx* gene expression in abdominal fat depots (Fig 6E) and higher plasma malondialdehyde levels, an oxidative stress marker in human (Fig 6F). Taken together, these results suggest that the subjects carrying risk NPGPx alleles may have reduced NPGPx expression in adipose tissue and higher BMI.

## DISCUSSION

NPGPx is an ER-resident oxidative stress transducer that transmits oxidative stress to downstream effecters, thereby alleviating cellular ROS (Nguyen et al, 2011; Peng et al, 2012; Utomo et al, 2004; Wei et al, 2012). Among all GPx member, NPGPx was mainly expressed in white adipose tissue. Depletion of NPGPx increased adipogenesis both *in vitro* and *in vivo* through ROS-dependent modulation of early transcriptional cascade for adipocyte differentiation. The increased ROS caused by NPGPx deficiency facilitated the dimerization of PKA regulatory subunits, leading to increased released of the PKA catalytic subunits and enhanced downstream signalling to activate C/EBPβ transcription.

It is of note that both the monomer and dimer forms of C/EBPβ were persistently higher in NPGPx-deficient cells after adipogenic stimulation. This can be attributed to the enhanced C/EBPβ transcription at the earlier time point. In addition, the ratio of C/EBPβ dimer to monomer was also increased in NPGPx-deficient cells during adipocyte differentiation. ROS is required for the disulphide bond formation between the leucine zipper regions of C/EBPβ to form a dimer, which the basic regions of two C/EBPβ monomers are brought together to create a ‘scissors-like’ DNA binding pocket to facilitate DNA binding (Kim et al, 2007). In NPGPx-deficient preadipocytes, increased ROS shifted the monomer-dimer equilibrium to dimer formation of C/EBPβ and hence facilitated adipocyte differentiation. Since many lipogenic enzymes such as fatty acid synthase, pyruvate kinase or ATP-citrate lyase have also been reported to harbour potential redox-sensitive cysteinyl residues (Weerapana et al, 2010; Ziegler, 1985), whether the increased ROS in NPGPx-deficient cells also modifies these downstream lipogenic enzymes to affect adipogenesis remains to be explored.

Cellular ROS is mainly generated from ER and mitochondria. It was reported that elevated ROS from mitochondria due to the loss of peroxiredoxin 3, a mitochondria-specific peroxidase, promoted fat accumulation in mice (Huh et al, 2012). This is consistent with our finding that deficiency of NPGPx, an ER-resident oxidative stress sensor/transducer, increases ROS and facilitates adipocyte differentiation as described herein. These results indicate that ROS either generated from ER or mitochondria may have an important role for adipocyte differentiation.

In addition to the activation of downstream lipogenic transcriptional cascade for terminal differentiation of adipocytes, C/EBPβ has a critical role for mitotic clonal expansion in early adipogenesis (Rosen & MacDougald, 2006; Tang et al, 2003). Consistently, in NPGPx-deficient cells, both mitotic clonal expansion in early adipogenesis and PPARγ expression in late differentiation were increased upon C/EBPβ activation. Similarly, increased cellular proliferation and PPARγ expression were also observed in adipose tissue in NPGPx knockout mice. These data indicate NPGPx deficiency promotes both adipocyte proliferation and hypertrophy.

Obesity results from the complex interaction between food intake, energy expenditure, and the tendency to deposit energy (so-called nutrient partitioning) (O'Rahilly, 2009). Although food intake was similar between NPGPx knockout and wild-type mice, energy expenditure in the dark/active phase and spontaneous ambulatory activity was reduced in knockout mice as compared to wild-type mice on HFD but not on chow. However, exercise capability was not impaired in these mice and we cannot detect any defect of mitochondrial oxidative phosphorylation, fatty acid oxidation or thermogenesis in these mice. Instead, a strong tendency to deposit energy as fat (*i.e*. enhanced adipogenesis potential) was observed in NPGPx knockout mice. These results suggest the obesity phenotype in NPGPx knockout mice was likely due to the enhanced adipogenesis.

The cause underlying the lower energy expenditure and spontaneous ambulatory activity in NPGPx knockout mice remained to be resolved. It is likely that adaptive changes of energy expenditure and spontaneous ambulatory activity may occur with increased adiposity. Interestingly, increased activity and energy expenditure was also observed in adipose-specific PPARγ knockout mice, which exhibited marked loss of fat mass and resistance to HFD-induced obesity due to defective adipogenesis (Jones et al, 2005), suggesting energy balance and activity level may be changed with altered adiposity. Alternatively, NPGPx deficiency may directly down-regulate energy expenditure through unknown mechanism, leading to the obesity. Future studies using tissue-specific NPGPx knockout mice will further strengthen this concept.

Interestingly, the *NPGPx* gene was previously mapped within a candidate quantitative locus for BMI (*p* = 0.009) by a genome-wide linkage analysis in human (Platte et al, 2003). A quantitative trait loci mapping for body weight in rats also identified a candidate region harbouring the *npgpx* gene (*p* = 0.01, LOD = 3.1) (Seda et al, 2005). We found that genetic variations near the *NPGPx* gene were associated with BMI in several independent human populations. These genetic variants are in strong LD with each other and rs835337 is the tagSNP for other SNPs at *r*^2^ of 0.8. The study-wide significance level that takes into account of the LD among SNPs was estimated to be 0.039 after correction of multiple markers (Nyholt, 2004). Nearly all *p*-values are lower than this threshold. Furthermore, a modest association of rs835337 and rs7529595 with BMI in the same direction was also found (*p* = 0.01 and 0.01, respectively) in the GIANT Consortium, a large meta-analysis of genome-wide association studies (GWASs) comprising 249,796 subjects (Speliotes et al, 2010). These data support a role of *NPGPx* in modulating BMI. Although how these SNPs in *NPGPx* gene affect BMI is currently unclear, the correlation between rs835337 genotype and *NPGPx* mRNA levels in human adipose tissue implicates that the functional variant may be involved in the regulation of *NPGPx* gene expression.

Importantly, the finding of the potent anti-adipogenic activity of NAC is particularly worth mentioning. NAC is a precursor of cysteine and can be converted intracellularly to the reduced form of glutathione and has been approved for the treatment of acetaminophen-induced hepatic injury and prevention of radiocontrast-induced nephropathy due to its potent anti-oxidant property (Heard, 2008; Marenzi et al, 2006). Our results clearly demonstrated that NAC effectively prevented fat accumulation through ROS scavenging (Fig 5). The mechanism by which NAC prevent adipogenesis *in vivo* was not fully elucidated. Previous studies proposed ROS suppresses food intake through modulation of hypothalamic melanocortin system and intracerebroventricular injection of ROS scavenger increased food intake (Diano et al, 2011). Our study, however, did not find significant effect of NAC on food intake. Specifically, NAC have no effect on weight in chow-fed mutant or wild-type mice, indicating limited drug toxicity. Interestingly, bardoxolone methyl, a novel antioxidant activating the Keap1-Nrf2 pathway, also showed unexpected weight-reducing effects in a recent clinical trial (Pergola et al, 2011). Similarly, alpha-lipoic acid, an ROS scavenger for hydroxyl radicals and singlet oxygen (Packer et al, 1995), exerts reproducible anti-obesity effects in several independent clinical trials (Carbonelli et al, 2010; Kim et al, 2008; Koh et al, 2011). The anti-adipogenic effect of NAC, an established and approved drug with a good safety profile, provides a new therapeutic opportunity for the prevention of obesity.

## MATERIALS AND METHODS

### Generation of NPGPx knockout mice

To generate NPGPx knockout mice, a gene-targeting strategy was used as previous described (Wei et al, 2012). To obtain homozygous litters, offspring with germline-transmitted targeted gene were obtained by backcrossing chimera mice to C57BL/6. NPGPx knockout (NPGPx^−/−^) mice and wild-type littermate (NPGPx^+/+^) were generated by heterozygous mating. Unless otherwise indicated, the mice used in these experiments were female. For gene expression profiling in epididymis, male mice were used.

### Animal protocols, diets and treatment

All mice were housed at 23°C and light/dark cycles of 12/12 h. All animal experiments were performed according to national ethical guidelines and were approved by the local animal experimentation committee of the Academia Sinica, Taiwan. Mice were fed *ad libitum* with a standard mouse chow diet (13% calorie from fat, TestDiet 5010) or HFD (60% calorie from fat, TestDiet 58Y1). For the NAC (Sigma A7250) treatment, NAC was added to drinking water at a concentration of 2 mg/1 mL and was renewed twice weekly.

### Micro-computed tomography (CT)

CT images were acquired using a microCT instrument (FLEX Triumph, General Electric) with mice under anaesthesia by inhalation of isofluorane in oxygen. The analysis of body fat mass was performed using the Amira software (Visage Imaging GmbH). Region of interest (ROI) comprising the trunk of mice was selected and the body fat was defined by a Hounsfield unit between −250 and −50.

### Indirect calorimetry

Mice were measured at the age of 18 weeks using an 8-chamber LabMaster Calorimetry Module (TSE-Systems GmbH) with one mouse per chamber. After acclimatization individually for 72 h, the O_2_ consumption (VO_2_, mL/kg/min), CO_2_ production (VCO_2_, mL/kg/min), and respiratory quotient (ratio of VCO_2_/VO_2_) were determined. VO_2_ and VCO_2_ were recorded every 30 min for a total of 48 h. The energy expenditure was calculated as the product of the calorific value of oxygen (3.815 + 1.232 × respiratory quotient) and VO_2_. To measure the food intake, mice were housed in metabolic cages for 72 h, and food and water intake were measured during the final 48 h.

### Serum metabolic parameters

Retro-orbital blood was collected after fasting for 6 h in mice at the age of 24 weeks under anaesthesia by inhalation of isofluorane in oxygen. Plasma triglyceride was quantified by the Fuji Dri-Chem Clinical Chemistry Analyzer. Plasma free fatty acids were measured using colorimetric assays (Wako 294-63601).

### Isolation of adipocytes and stromal vascular fraction (SVF) from white adipose tissue

Four week-old female mice were euthanized by CO_2_ inhalation, and inguinal fat pads were collected, minced and digested with type I collagenase at 37°C in a shaking water bath for 60 min. The digested fat was filtered through a sterile 70 µm nylon mesh to remove undigested fragments. The filtered suspension was centrifuged at 300 g for 10 min. Floating cells and the cell pellet were recovered as the mature adipocyte fraction and the SVF, respectively. Each fraction was washed twice with PBS. The SVF was incubated in erythrocytes lysis buffer (155 mM NH_4_Cl, 10 mM KHCO_3_ and 0.1 mM EDTA) for 10 min, centrifuged to pellet and washed with PBS. The SVF was then cultured in Dulbecco's modified Eagle medium (DMEM) Nutrient Mixture F-12 (Invitrogen 11320-033) with 5% fetal bovine serum (FBS). When the cells reached confluence, differentiation was induced by adding adipogenic differentiation medium.

### Fluorescence-activated cell sorting (FACS) analysis for determination of intracellular ROS

For ROS measurement, primary SVF and adipocytes isolated from gonadal fat pads were incubated with CM-H_2_-DCFDA (5 µM) (Molecular Probe C6827, Invitrogen) in phenol-free DMEM. CM-H_2_-DCFDA is an ester probe that reacts with intracellular ROS and the oxidative adduct of CM-H_2_-DCFDA emits green florescence upon excitation. After 60 min of incubation, cells were analysed by FACS Canto II instrument (BD Biosciences) using excitation at 485 nm and emission at 530 nm. The mean fluorescence intensity was used to estimate cellular ROS levels.

### *In vitro* ROS measurements in adipose tissue

Gonadal fat pads were stored in liquid nitrogen immediately after harvest. Fat pads (about 100 mg) were homogenized in 1 mL of PBS, sonicated, and centrifugated in 4°C to removed debris and floating lipid. The ROS amount of homogenate was determined using commercial kit (Cell Biolabs STA347) according to manufacturer's instruction. Briefly, the quenched DCF-DiOxyQ probe was primed to DCF-DiOxy and then converted to the non-fluorescent DCFH probe. DCFH can react with ROS to form fluorescent DCF. Tissue homogenate was then incubated with DCFH and catalytic agents. The fluorescence intensity was analysed using excitation at 485 nm and emission at 530 nm and was normalized to the total protein content of tissue homogenates.

### Plasmids

Small hairpin RNA (shRNA) plasmids for the mouse *NPGPx* gene and the empty control vector (pLKO_TRC014) were obtained from the National RNAi Core Facility (Academia Sinica, Taiwan) and the target sequences for mouse NPGPx was 5′-CCTTCGGAAACGAGAAGACTT-3′. Human NPGPx cDNA was inserted in lentiviral vectors (pLKO-AS2) containing a hygromycin selection marker. Lentivirus production and infection of cells were performed following the procedures of the National RNAi Core Facility.

### Nitroblue tetrazolium (NBT) reduction assay

Intracellular ROS level was determined by the NBT reduction assay. Cells were harvested at the times indicated and incubated with 0.2% NBT (Sigma N6876) for 90 min. After incubation, the reduced NBT was dissolved in 50% acetic acid and the absorbance was determined at 560 nm.

### Electrophoretic mobility shift assay (EMSA)

Nuclear protein from differentiated 3T3-L1 cells were extracted using the ProteoJET Cytoplasmic and Nuclear Protein Extraction Kit (ThermoFisher Scientific 0311) and EMSA was performed using the LightShift Chemiluminescent EMSA Kit (Pierce 20148). The nuclear extract was incubated for 30 min at room temperature with a biotinylated oligonucleotide containing the C/EBP β binding site of the C/EBPα promoter 5′-GCGTTGCGCCACGATCTCTC-3′. For competition experiments, nuclear extracts were incubated with an unlabelled competitor oligonucleotide (100-fold molar excess) for 15 min before the addition of the biotin-labelled probe. After the binding reaction, the mixture was separated electrophoretically on a 6% polyacrylamide gel in 0.5 × TBE and was then transferred to nylon membrane. The biotinylated oligonucleotide probe was detected by chemiluminescence using the Chemiluminescent Nucleic Acid Detection Module (Pierce 89880).

### Histology, adipocyte size measurement and adipocyte number estimation

For haematoxylin and eosin (HE) staining, white adipose tissues was fixed in 4% paraformaldehyde, processed and embedded in paraffin before sectioning and staining. Two sections were obtained for each mouse. The stained sections were scanned and analysed using MIRAX Viewer (http://www.zeiss.de/mirax) and Image J software (http://rsbwed.nih.gov/ij/). For adipocyte size measurements, 200 consecutive fat cells of the gonadal fat pad from each mouse were selected for the area measurement. The adipocyte number of the gonadal fat pad was calculated as the fat pad volume divided by the average fat cell volume. Fat pad volume was calculated as fat pad weight (g) divided by fat density (0.915 g/cm^3^).

### Cell proliferation assay

The quantification of cell proliferation was performed using a commercial kit (Roche 11669915001). 3T3-L1 cells were treated with induction medium for 18 h with or without NAC and were labelled with bromodeoxyuridine (BrdU). Cells were then fixed, stained with anti-BrdU antibody and visualized using a chemiluminescent immunoassay.

### Protein extraction and Western blotting

Total protein from cells was extracted with RIPA buffer [50 mM Tris–HCl, pH 7.4, 150 mM NaCl, 2 mM ethylenediaminetetraacetic acid (EDTA), 50 mM NaF, 1% Nonidet P-40, 0.5% sodium deoxycholate, 0.1% sodium dodecyl sulphate (SDS) and 1 mM phenylmethylsulphonyl fluoride (PMSF)] for 5 min, sonicated, and centrifuged to removed debris. For reducing SDS-polyacrylamide gel electrophoresis, the protein sample was mixed with SDS sample buffer (containing dithiothreitol, DTT) and denatured by boiling for 5 min. For non-reducing SDS-polyacrylamide gel electrophoresis, the protein sample was reconstituted in non-reducing SDS sample buffer (without DTT). Primary antibodies targeted NPGPx (ProteinTech 13501), aP2 (R&D AF1443), adiponectin (Biovision 5900), α-tubulin (GeneTex 11302), GAPDH (GeneTex 627408), C/EBPβ (BioLegend 606201), PKA C-α (Cell Signalling 4782), PKA-RI-α (Cell Signalling 5675), phospho-CREB (Millipore 05-667) and CREB (Cell Signalling 9104) was used.

### Cell culture

The 3T3-L1 preadipocytes were cultured in high-glucose DMEM (Invitrogen 11965) with 10% calf serum at 37°C in an atmosphere of 5% CO_2_. Post-confluent cells were induced by adipogenic differentiation medium containing high-glucose DMEM, 10% FBS (GibcoBRL 16170), 1 µM dexamethasone (Sigma D4902), 0.5 mM isobutylmethylxanthine (Sigma I7018) and 5 µg/mL of bovine insulin (Sigma I5500). After 2 days, the cells were maintained in high-glucose DMEM containing 10% FBS and 5 µg/mL of bovine insulin. After another 2 days, medium was changed to high-glucose DMEM containing 10% FBS until the day 8 harvest. Cells were stained with Oil Red O (Sigma O0625) to measure the degree of adipocyte differentiation. To assess the effect of NAC, tiron (Sigma 89460) or PBA (Merck4Bioscineces 567616) on adipocyte differentiation, these reagents were added at the first 2 days after the induction with the indicated concentration, respectively. For NAC effect on PKA regulatory subunit dimerization, cells were pretreated with NAC for 24 h.

### Quantitative reverse transcriptase PCR (qRT-PCR)

Total RNA was extracted from respective tissues or cells using the TRIzol (Life Technologies) according to the manufacturer's instructions and was reverse-transcribed into cDNA using commercially available kits (Roche 11483188001). All subsequent quantitative qRT-PCR reactions were performed using the Applied Biosystems 7300 Real-Time system (Applied Biosystems) with the SYBR Green dye (Roche 04913850001). The threshold cycles (Ct-values) of all triplicates were normalized to 1*8S rRNA* within each sample to obtain the sample-specific ΔCt values. To compare the effect of various treatments, ΔΔCt values were calculated. All other primers used are listed in Supporting Information Table S2.

### Genetic association studies: SNP selection, clinical data collection and genotyping platform

We first searched the GWAS Central database (https://www.gwascentral.org) for association with BMI near/within the *NPGPX (GPX7)* gene using *p*-value threshold of 0.05 and 10 kb-flanking region as search criteria. Three SNPs (rs835337, rs7529595, rs6588432) were identified in the British 1958 Birth Cohort study. These three SNPs were then analysed for replication in the North Finland 1966 Birth Cohort study and were further genotyped in Han Chinese replication sample.

The British 1958 cohort includes all births in England, Wales and Scotland, during one week in 1958. This cohort was originally recruited for a perinatal mortality and morbidity survey, and the participants were monitored throughout the school years with medical examinations. In this present study, blood samples were collected from 1424 cohort members at age 44–45. Genotyping was performed on Affymetrix GeneChip Mapping 500K, Custom Illumina Infinium and Illumina Infinium HumanHap550 genotyping platform. Detailed genotype information was retrieved from data deposited by Dr. Panos Deloukas (Wellcome Trust Sanger Institute).

The Northern Finland Birth Cohorts of 1966 (NFBC 1966) includes 12,058 live births to mothers in the two northern-most provinces of Finland. Follow-up of the NFBC 1966 with clinical data collection was performed at the age of 31 with assessment of a wide range of traits. DNA samples were obtained from 5923 subjects of NFBC1966. The GWAS was conducted in 4763 subjects using Illumina Human CNV370 v1 genotyping platform. A detailed description of the NFBC1966 GWAS can be found elsewhere (Sabatti et al, 2009). Detailed genotype and phenotype association analysis was requested from Dr. Freimer, Nelson B (University of California, LA).

Seven hundred and sixty Han Chinese subjects were recruited from a community-based screening program for the metabolic syndrome in Taiwan (Lin et al, 2009). Exclusion criteria were as follows: age < 18 years, pregnant women, previously diagnosed diabetes or previously diagnosed major systemic diseases. Genomic DNA was extracted from peripheral blood leukocyte. Genotyping was performed using the Taqman platform (Life Technologies). Body weight, height and waist circumference was measured by trained nurse. Fasting plasma was collected after overnight fasting. Plasma malodialdehyde was measured by reaction with thiobarbituric acid (TBA) (Biovision K739-100). The Institutional Review Board of the National Taiwan University Hospital Research Ethics Committee approved this study. Written informed consent was obtained from each participant.

### Gene expression in abdominal fat depots

We recruited 84 female adults undergoing bariatric surgery or elective abdominal surgery such as cholecystectomy or partial hepatectomy from Ming-Sheng General Hospital in Taiwan. Abdominal subcutaneous fat depots were sampled in a fasting state during surgery and were placed in liquid nitrogen immediately until processing. The study was approved by the institutional review board of Ming-Sheng General Hospital and National Taiwan University Hospital. Written informed consent was obtained from each patient.

### Statistical analyses

Independent Student's *t*-tests were used for comparisons between two independent groups. Paired *t*-tests were used to compare mRNA levels in fat pads from littermate mice pairs. The correlation between two groups was analysed using Pearson's correlation. The comparisons of body weight between two groups over time were analysed using repeated measure ANOVA. Non-parametric trend test for trend was used analysed the association of *NPGPx* gene expression levels across different genotypes. A Hardy–Weinberg equilibrium test was performed for each sequence variant before the marker-trait association analysis. The inter-marker LD was measured by pairwise D′ and *r*^2^ using the Haploview software (Barrett et al, 2005). Study-wide significance level taking into account of the LD between SNPs to keep a type 1 error of 5% for multiple testing was determined using the Nyholt's procedure (Nyholt, 2004). Linear regression in additive model was used to analyse the association between SNP and BMI in the British 1958 Birth Cohort and the Northern Finland Birth Cohorts of 1966. Linear regression in additive, recessive, and dominant models were used to analyse SNP association with BMI in the Han Chinese replication sample. The maximum tests were used to adjust *p*-value for multiple genetic models (So & Sham, 2011). Statistical analyses were conducted using SAS 9.0 and STATA 10.0. Meta-analysis was conducted using inverse-variance method for fixed effects implemented in the Comprehensive Meta-analysis (CMA) software. Cochran's Q and I square were used to measure the heterogeneity between studies. All *p*-values < 0.05 were considered as statistically significant.

Obesity is serious global epidemic with huge health impact. Elevated oxidative stress is often observed in obese subjects and considered as a consequence of excessive fat accumulation. However, emerging evidence suggest that oxidative stress may contribute to adipogenesis. We have identified NPGPx as a sensor and transducer for oxidative stress signal and also found that NPGPx was mainly expressed in white adipose tissue. We ask whether NPGPx play an essential role in fat metabolism.

RESULTS:

We found that deficiency of NPGPx raised ROS level in preadipocytes and promoted adipocyte differentiation via increasing oxidative stress. The increased oxidative stress causes conformational change of protein kinase A regulatory subunits, leading to activation of C/EBPβ, a critical transcriptional factors for adipogenesis. Consistently, NPGPx-deficient mice exhibited markedly increased fat mass and adipocyte hypertrophy. Treatment with antioxidant NAC ablated these phenotypes. Furthermore, genetic variation in human *NPGPx* gene correlated with lower NPGPx expression level in adipose tissues, were associated with higher body mass index in several independent human populations.

IMPACT:

NPGPx is an important drug target as well as a biomarker for obesity. Patients carrying certain obesity-prone NPGPx genetic variant may benefit from antioxidant therapy.

## Author contributions

YCC and YHY conducted experiments, analyzed data and wrote the manuscript. JYS coordinated all experiments and animal breeding and housing. WJL provided adipose tissue samples. JJH provided Han Chinese DNA samples and clinical data. YHC performed exercise endurance test. YRC performed lipidomics study. PCW provided plasmids. LMC and WHL designed the experiments, interpreted data and wrote the manuscript.

## Supporting Information

As a service to our authors and readers, this journal provides supporting information supplied by the authors. Such materials are peer reviewed and may be re-organized for online delivery, but are not copy-edited or typeset. Technical support issues arising from supporting information (other than missing files) should be addressed to the authors.
