# Supplementary material for: Deficiency of NPGPx, an oxidative stress sensor, leads to obesity in mice and human
Source: EMBO Mol Med. 2013 Jul 4;5(8):1165–79. doi: 10.1002/emmm.201302679 (PMC3944459; doi:10.1002/emmm.201302679)
Supplement: Supplementary file 1 [file emmm0005-1165-SD1.pdf]

Deficiency of NPGPx, an oxidative stress sensor, leads to obesity in mice and human

Yi-Cheng Chang, Yu-Hsiang Yu, Jin-Yu Shew,Wei-Jei Lee, Juey-Jen Hwang, Yen-Hui Chen, Yet-Ran Chen, Pei-Chi Wei, Lee-Ming Chuang, Wen-Hwa Lee

Corresponding authors: Wen-Hwa Lee, Dept. of Biological Chemistry, University of California and Yi-Cheng Chang, Genomics Research Center, Academia Sinica

Review timeline:

Submission date:	26 February 2013
Editorial Decision:	20 March 2013
Revision received:	10 May 2013
Editorial Decision:	29 May 2013
Revision received:	30 May 2013
Accepted:	03 June 2013

Transaction Report:

(Note: With the exception of the correction of typographical or spelling errors that could be a source of ambiguity, letters and reports are not edited. The original formatting of letters and referee reports may not be reflected in this compilation.)

Editor: Roberto Buccione

1st Editorial Decision 20 March 2013

Thank you for the submission of your manuscript to EMBO Molecular Medicine. We have now received reports from the three Reviewers whom we asked to evaluate your manuscript.

You will see that while the Reviewers are generally supportive, they all raise critical points that question the impact and conclusiveness of the results, thus preventing us from considering publication at this time.

Reviewer 1's main concern is on the modest associations found in your human studies and suggests that you provide a metanalysis of the available studies showing association of NPGPx and obesity. Reviewer 1 also lists other issues, which also require your action.

Reviewer 2, who is an expert in SNP association analysis, notes that insufficient information is provided on your SNP association analysis and raises doubts concerning significance and asks you to provide additional data to this effect or re-run the analysis appropriately.

Reviewer 3 is more supportive but points to the unclear causal relationship between the in vitro observations and the in vivo findings while noting a possible contradiction between your interpretation and the known impact of increased adipogenesis on metabolic health. To this effect s/he suggest a number of experimental approaches to resolve the issue.

While publication of the paper cannot be considered at this stage, we would be prepared to consider a suitably revised submission, with the understanding that the Reviewers' concerns must be fully

addressed with additional experimental data, where appropriate together with the provision of a full, detailed description of the SNP analysis (for instance in the supplementary information). Your revised the manuscript will undergo a second round of review.

Please note that it is EMBO Molecular Medicine policy to allow a single round of revision only and that, therefore, acceptance or rejection of the manuscript will depend on the completeness of your responses included in the next, final version of the manuscript.

As you know, EMBO Molecular Medicine has a "scooping protection" policy, whereby similar findings that are published by others during review or revision are not a criterion for rejection. However, I do ask you to get in touch with us after three months if you have not completed your revision, to update us on the status. Please also contact us as soon as possible if similar work is published elsewhere.

I look forward to seeing a revised form of your manuscript as soon as possible.

***** Reviewer's comments *****

Referee #1 (Comments on Novelty/Model System):

It is already demonstrated by the authors that NPGPx is essential for releasing excessive ER stress to maintain physiological homeostasis. In this manuscript, Chang et al. provide evidences according to which NPGPX deficiency is associated with development of obesity.
The models studied are consistent, and the way which authors use to rescue the phenotype is compelling.
I have the following comments:
NPGPx is increased in SVCs and then decrease during differentiation. In addition to preadipocytes, other cells are present in this SVC fraction, potentially important in the development of hypertrophied adipose tissue. What about NPGPX expression in macrophages or endothelial cells?

The weakest part of the manuscript is the human genetic studies. Only a modest association was found in the GIANT Consortium (p=0.01). GIANT usually employs a log-additive model. Was this consistent in the other populations?
The p value in Han Chinese adults is even lower (p=0.03 with waist circumference).
The authors should provide a meta-analyses of all available studies showing that the genetic variants of NPGPX are associated with obesity.

Pages are not numbered.
There is a typo in page 8: adsipocyte

Referee #2 (General Remarks):

This paper investigated the NPGPx is highly expressed in preadipocytes but not in mature adipocytes of white adipose tissue. They further showed that SNPs near the NPGPx gene were associated with increased adiposity in several human populations.

I have some comments on the SNP association analysis of the paper:

1. It is not clear how many SNPs were tested in the analysis. The paper only mentioned 3 SNPs, rs835337m, rs7529595 and rs6588432. However, with GWAS data, the authors may have also looked at other SNPs located near or within GPX7. If so, what is the exact number of SNPs that were tested? This is important as it relates to the multiple testing issue. If a large number of SNPs were tested, then using 0.05 as the p-value threshold for declaring significance is not appropriate.

2. Another related issue is that for each SNP, the authors conducted 3 tests assuming additive, dominant and recessive models. There is no mentioning on how multiple testing is corrected.

3. The smallest SNP association p-value is 0.005 (from additive model for rs835337). But since at least 3 SNPs were tested, and each SNP was tested for additive, dominant and recessive effect, the conservative p-value threshold for declaring significance would be 0.05/(3x3) = 0.0056. If the authors tested more than 3 SNPs, then the p-value threshold would be even lower and rs835337 may no longer significant if multiple testing is appropriately corrected.

Referee #3 (Comments on Novelty/Model System):

The manuscript is of high importance as it details an important mechanism (ROS signalling) that has gained a lot of attention in the last few years. While it was thought for a long time that ROS production is negative, it is clear now that ROS signalling is needed to drive certain biological processes such as muscle cell formation or in this case adipocyte formation. The problem I see is the bridge between the nice molecular and the nice physiological data presented here.

Referee #3 (General Remarks):

The paper entitled "Deficiency of NPGPX, an oxidative stress sensor, leads to obesity in mice and human" by Chang and colleagues describes the regulation of adipocyte formation in light of ROS scavenging. The authors show in several beautiful experiments that NPGPX controls ROS formation and that loss of this gene induces adipocyte formation through regulation of CREB and CEBPb activity, one major hub in adipogenesis. In addition they show that NPGX mice develop obesity on a high fat diet concomitant with an increase in adipocyte size. Lastly, the authors show that a SNP in NPGPX is associated with obesity in several cohorts which suggests that this finding can be translated from mouse to humans.

Both parts themselves are very well done, the problem I see is that the in vitro finding cannot explain the in vivo findings. Obesity cannot develop just because more adipocytes are formed. For obesity to develop there would have to be shift in energy balance. Actually quite to the contrary, it can be expected that increased adipogenesis, without an alteration in energy expenditure would improve metabolic health (see glitazone action) as adipocytes would store lipids better and thus reduce circulating lipids. The authors here actually report the opposite as adipocyte size is changed while adipocyte number is only marginally increased. Some ideas that could reconcile this problem are the following:

1. The metabolic cage data is measured after 12 weeks of HFD, maybe a measurement of the mice before HFD reveals a difference that is masked by the HFD effect.
2. Is the body temperature of the animals changed
3. Would increased ROS levels in the SVF elicit any kind of response that could lead to alterations in the energy balance, inflammation comes to mind.
4. Is in vivo adipogenesis affected

Alternatively, it could well be that another tissue contributes to the observed phenotype which is difficult to prove as there are no usable mouse lines to generate a pre-adipocyte specific knock out mouse.

Minor points: I would switch Figure 2 with 3 and 4, as this would improve the flow of the manuscript.

1st Revision - authors' response 10 May 2013

Attached please find the revised manuscript (EMM-2013-02679) entitled “Deficiency of NPGPx, an oxidative stress sensor, leads to obesity in mice and human“ by Yi-Cheng Chang et al. for your consideration of publishing in EMBO Molecular Medicine. We thank these three reviewers for their encouragements of this interesting work and appreciate their thoughtful suggestions. Based on their comments, we have accordingly performed several additional experiments and SNP analysis to

address all their concerns. I have briefly summarized our responses as follows. In addition, the detailed point-by-point response to the comments is also attached.

Reviewer 1's main concern is on the modest associations found in your human studies and suggests that you provide a metanalysis of the available studies showing association of NPGPx and obesity. Reviewer 1 also lists other issues, which also require your action.

Answer: In the revised manuscript, we performed a meta-analysis pooling all available studies that shows the significant associations between NPGPx SNPs and BMI. In addition, we have analysed the NPGPx expression level in different type of cells including macrophages, endothelial cells, and preadipocytes in adipose tissue and found the preadipocytes contain the highest level.

Reviewer 2, who is an expert in SNP association analysis, notes that insufficient information is provided on your SNP association analysis and raises doubts concerning significance and asks you to provide additional data to this effect or re-run the analysis appropriately.
Answer: We provided the detailed information of SNP analysis (the number of SNPs and statistical analyses performed). The significance levels of association between NPGPx SNP and BMI were further adjusted for multiple markers and multiple genetic models. Similarly, significant association between NPGPx SNPs and BMI was confirmed using meta-analysis as addressed to the reviewer 1.

Reviewer 3 is more supportive but points to the unclear causal relationship between the in vitro observations and the in vivo findings while noting a possible contradiction between your interpretation and the known impact of increased adipogenesis on metabolic health. To this effect s/he suggested a number of experimental approaches to resolve the issue.
Answer: We appreciate very much the reviewer's comment and have performed additional experiments showing that deficiency of NPGPx enhanced adipogenesis both in vitro and in vivo. We further found long-term excessive fat accumulation in NPGPx-deficient mice on high-fat diet causes macrophage infiltration and inflammatory changes in adipose tissue, which may promote hyperlipidemia and insulin resistance. This explains, in part, the observed phenotypes.

We believe that the current revised manuscript has met the high standard for publication in EMBO Molecular medicine. We greatly appreciate your time in serving as the editor handling this manuscript.

Detailed point-by-point response to reviewers' comments

Referee #1:
It is already demonstrated by the authors that NPGPx is essential for releasing excessive ER stress to maintain physiological homeostasis. In this manuscript, Chang et al. provide evidences according to which NPGPX deficiency is associated with development of obesity. The models studied are consistent, and the way which authors use to rescue the phenotype is compelling.

Answer: Thank you for the kind encouragement.

Comments:
(1) NPGPx is increased in SVCs and then decrease during differentiation. In addition to preadipocytes, other cells are present in this SVC fraction, potentially important in the development of hypertrophied adipose tissue. What about NPGPX expression in macrophages or endothelial cells?

Answer: To examine the expression of NPGPx in subpopulations in the stromovascular (SVF) fraction, we isolated preadipocytes ($Lin^{-}CD34^{+}CD29^{+}Sca\text{-}1^{+}$), endothelial cells ($CD31^{+}$), monocytes/macrophages ($CD11b^{+}$) using specific cell surface markers and adipocytes from adipose tissue of C57BL/6 mice and measured NPGPx gene expression by RT-qPCR As shown in Fig. R1 (also shown in Fig. 1B in revised manuscript), NPGPx was highly expressed in preadipocytes as compared to macrophages, endothelial cells, and mature adipocytes.

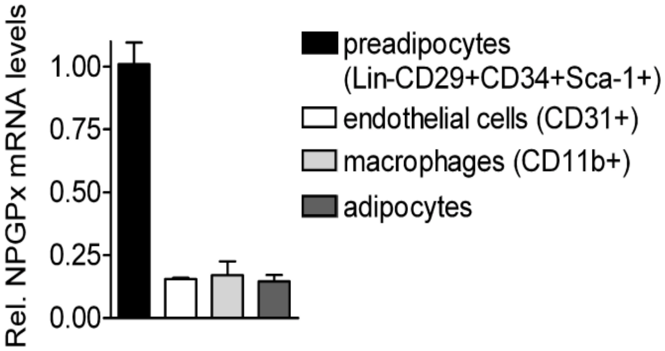

Fig. R1. Relative NPGPx mRNA levels in preadipocytes, endothelial cells, macrophages, and adipocytes in white adipose tissue. Each lineage of cells was isolated from the white adipose tissues of C57BL/6 mice using specific surface markers. The relative amount of NPGPx mRNA to 18S rRNA was measured by RT-qPCR. The experiment was repeated three times.

(2) The weakest part of the manuscript is the human genetic studies. Only a modest association was found in the GIANT Consortium (p=0.01). GIANT usually employs a log-additive model. Was this consistent in the other populations?

Answer: Indeed, the association is only modest in the GIANT consortium. However, the association remains significant for a candidate-gene association analysis of rs835337 and rs7529595, respectively in the GIANT Consortium (P=0.01 and 0.01). The direction of this association (the minor alleles of these SNPs are associated with lower BMI) was also consistent with the results of our study as shown below (Fig R2).

(3) The p value in Han Chinese adults is even lower (p=0.03 with waist circumference).

Answer: Indeed, the association of rs835337 with waist circumference in Han Chinese samples was marginally significant due to the small population size. Since the waist circumference was analysed only in Han Chinese samples but not in other human samples, we decided to focus all analyses on the association with BMI throughout this study for consistency. Thus, we decided to remove this data from the manuscript to avoid confusion.

(4) The authors should provide a meta-analyses of all available studies showing that the genetic variants of NPGPX are associated with obesity.

Answer: A meta-analysis pooling all 3 available studies (North Finland Birth Cohort 1966, British 1958 Birth Cohort, and Han Chinese) comprising a total of 6,969 subjects shows significant associations between body mass index (BMI) and *NPGPx* SNPs (P=0.0004, 0.002, and 0.0003, respectively for rs835337, rs7529595, and rs6588432). The effect size is consistent across all 3 studies with per-minor allele decrease in BMI (in Z score) being 0.057, 0.115, and 0.082, respectively (pooled effect size= 0.074, 95% CI =0.033-0.115). There is no significant heterogeneity among studies (Cochran's Q=1.32, 1.91, 0.82 with P=0.49, 0.63, 0.66 and I square=0 %, 0 %, 0 %, respectively) (Fig R2, also see Supporting Information Fig. S11 and page 10, line 11-13 in the revised manuscript).

© EMBO

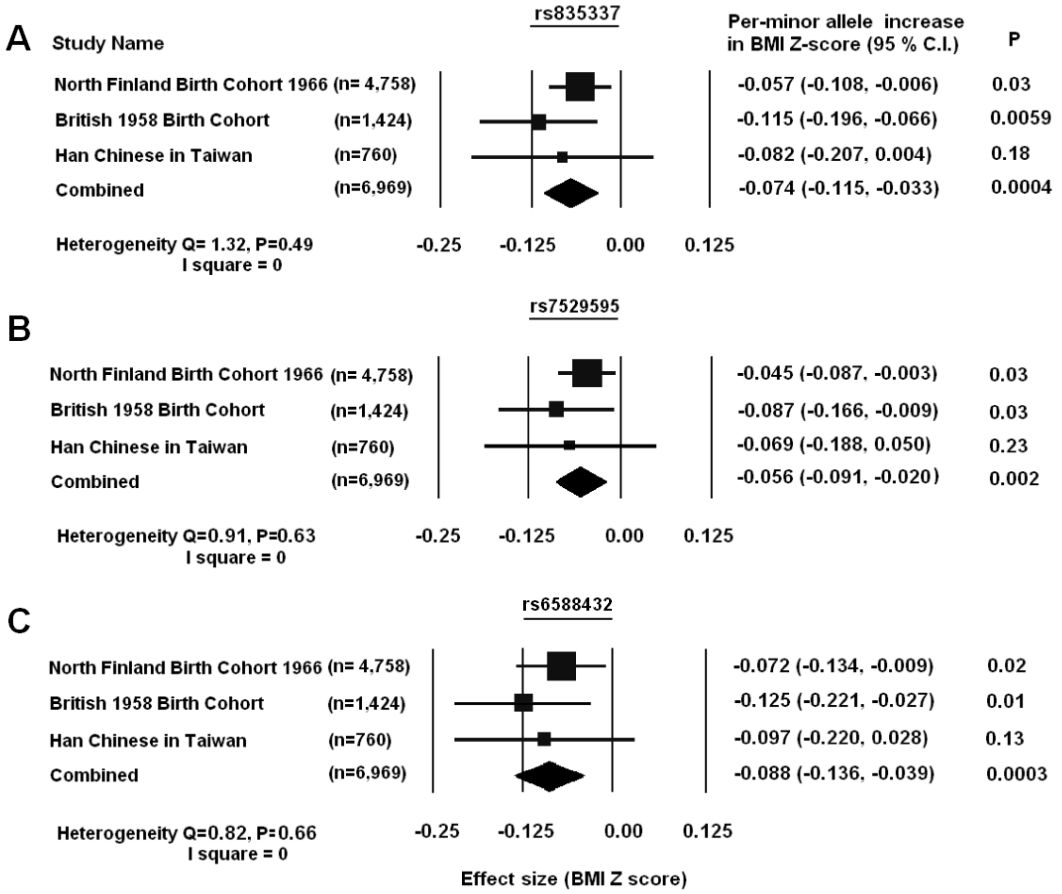

Fig. R2 Forest plots for meta-analyses of the association between body mass index (BMI) and SNPs near/within the *NPGPx* gene, including (A) rs835337, (B) rs7529595, and (C) rs6588432. Estimated per-minor allele effects on BMI are shown in Z score.

(3) Pages are not numbered. There is a typo in page 8: adsipocyte

Answer: Thank you very much! We have corrected these errors in revised manuscript (please see page 8, line 2 in the revises manuscript).

Referee #2:

This paper investigated the NPGPx is highly expressed in preadipocytes but not in mature adipocytes of white adipose tissue. They further showed that SNPs near the NPGPx gene were associated with increased adiposity in several human populations.
I have some comments on the SNP association analysis of the paper:
(1) It is not clear how many SNPs were tested in the analysis. The paper only mentioned 3 SNPs, rs835337, rs7529595 and rs6588432. However, with GWAS data, the authors may have also looked at other SNPs located near or within GPX7. If so, what is the exact number of SNPs that were tested?

Answer: We thank the reviewer for this important consideration. For initial SNP selection, we first searched the GWAS Central database (https://www.gwascentral.org) for SNP association with BMI near/within the *GPX7* gene using *P*- value threshold of 0.05 and 10 kb-flanking region as search criteria. Three SNPs (rs835337, rs7529595, rs6588432) were identified in the British 1958 Birth Cohort study. These 3 SNPs were in strong LD with each other and rs83577 is the tagSNP for other 2 SNPs at r^2 of 0.8 (please also see response below). We then requested analysis of these 3 SNPs for

replication in the North Finland 1966 Birth Cohort study (data from Dr. Freimer, Nelson B, UCLA). These 3 SNPs were further genotyped in the Han Chinese sample for replication. The detailed information is now provided in methods of the revised manuscript (pleas see page 20, line 9-13).

(2) This is important as it relates to the multiple testing issue. If a large number of SNPs were tested, then using 0.05 as the p-value threshold for declaring significance is not appropriate.

Answer: We fully agree with the reviewer that correction for multiple markers is required. These 3 SNPs are located within a large linkage disequilibrium (LD) block spanning the entire *NPGPx* gene and are in strong LD with each other (D'=1 and r^2=0.82 between rs835337 and rs7529595; D'=1 and r^2=0.94 between rs835337 and rs6588432; D'=0.99 and r^2=0.98 between rs7529595 and rs6588432). According to the correction method by Dale R. Nyholt (reference 1, online tool: http://genepi.qimr.edu.au/general/daleN/SNPSpD/), testing for these 3 correlated SNPs are equivalent to testing 1.28 independent SNPs after taking into consideration of LD between each other, which means the study-wide significance level to keep type I error rate at 5% is 0.039 (0.05/1.28). Bonferroni correction is usually thought to be too stringent under this condition (reference 1, 2). We have added this information in the revised manuscript (page 13, line 8-11 and page 22, line 3-5).

(2) Another related issue is that for each SNP, the authors conducted 3 tests assuming additive, dominant and recessive models. There is no mentioning on how multiple testing is corrected. The smallest SNP association p-value is 0.005 (from additive model for rs835337). But since at least 3 SNPs were tested, and each SNP was tested for additive, dominant and recessive effect, the conservative p-value threshold for declaring significance would be 0.05/(3x3) = 0.0056. If the authors tested more than 3 SNPs, then the p-value threshold would be even lower and rs835337 may no longer significant if multiple testing is appropriately corrected.

Answer: We fully appreciate the suggestion. Only additive model was tested in the North Finland 1966 and British 1958 Birth Cohort studies because the statistical analysis for these 2 studies was obtained through request and only analysis in additive model was provided the original investigators. Therefore, we did not adjust *P*-values for multiple genetic models in these two studies. All *P*-values in these two studies pass the study-wide significance level (*P*=0.039 by Nyholt's procedure) (please also see Fig. R2 in the reply to reviewer 1, Supporting Information Fig. S11 in the manuscript, and page 22, line 5-7).

We tested 3 genetic models (additive, recessive, and dominant) in the Han Chinese replication samples because the genotyping was performed by ourselves and we had a full access to the data. We agree with the reviewer that testing in multiple genetic models results in increased type 1 error and correction for multiple genetic models is required. Since test statistics under the additive, recessive, and dominant genetic models are not completely independent, Bonferroni correction is usually thought to be too conservative for correction of correlated multiple genetic tests (reference 3, 4, 5). Several approaches such as the maximum tests (MAX) are commonly used to adjust *P*-value over correlated genetic models (reference 5, online tool: http://sites.google.com/site/honcheongso/software/robustsnp). The MAX-adjusted *P*-values over 3 genetic models are estimated to be 0.008, 0.036, and 0.009 for rs835337, rs7529595 and rs6588432, respectively in Han Chinese replication sample. All these *P*-values are lower than the study-wide threshold (*P*=0.039 by Nyholt's procedure). Even when stringent Bonferroni correction was applied for correction of multiple genetic models, two of the 3 corrected *P*-values in Han Chinese population [0.015 (=0.005 x 3), 0.056 (=0.018 x 3), 0.024 (=0.008 x 3) for rs835337, rs7529595, and rs6588432] remain lower than the study-wide threshold (*P*=0.039). (please also see page 10, line 12-13 and page 22, line 7-9).

Importantly, the gold standard for validation of genetic association is replication in additional independent cohorts (reference 6). Although the initial SNP association may be a false-positive finding that arose from multiple testing, repeated associations of these SNPs with BMI were observed in independent human samples. Furthermore, a meta-analysis pooling all available studies (North Finland 1966 Cohort, British 1958 Birth Cohort study, and Han Chinese samples) showed very significant association of these SNPs with BMI (pooled *P*-values: 0.0004, 0.002, and 0.0003 for rs835337, rs7529595, and rs6588432, respectively), further supporting our conclusion (please see Fig. R2 shown in response to Reviewer 1 and Supporting Information Fig. S11).

Referee #3:

The manuscript is of high importance as it details an important mechanism (ROS signalling) that has gained a lot of attention in the last few years. While it was thought for a long time that ROS production is negative, it is clear now that ROS signalling is needed to drive certain biological processes such as muscle cell formation or in this case adipocyte formation. The problem I see is the bridge between the nice molecular and the nice physiological data presented here.

Referee #3 (General Remarks):
The paper entitled "Deficiency of NPGPX, an oxidative stress sensor, leads to obesity in mice and human" by Chang and colleagues describes the regulation of adipocyte formation in light of ROS scavenging. The authors show in several beautiful experiments that NPGPX controls ROS formation and that loss of this gene induces adipocyte formation through regulation of CREB and CEBPb activity, one major hub in adipogenesis. In addition they show that NPGPX mice develop obesity on a high fat diet concomitant with an increase in adipocyte size. Lastly, the authors show that a SNP in NPGPX is associated with obesity in several cohorts, which suggests that this finding can be translated from mouse to humans.

Answer: We thank the reviewer for these encouraging comments.

1. Both parts themselves are very well done, the problem I see is that the in vitro finding cannot explain the in vivo findings. Obesity cannot develop just because more adipocytes are formed. For obesity to develop there would have to be shift in energy balance.

Answer: We are fully aware of this point. Obesity results from the complex interaction between food intake, energy expenditure, and the tendency to deposit energy (so-called nutrient partitioning) (reference 7). Although food intake is similar between NPGPx knockout and wild-type mice, energy expenditure in the dark/active phase was reduced in knockout mice on HFD as compared to wild-type mice (Fig. R4). Spontaneous ambulatory activity was also decreased in these mice (Fig. R5A). However, the exercise capability was not altered in these mice. Furthermore, we cannot detect any defect in exercise capability, mitochondrial oxidative phosphorylation, fatty acid oxidation or thermogenesis (Fig. R5B-5G) in NPGPx knockout mice. Instead, a strong tendency to deposit energy as fat (i.e., enhanced adipogenesis potential) was observed in NPGPx knockout mice (Fig. R6). These results suggest the obesity phenotype in NPGPx knockout mice was likely due to the enhanced adipogenesis. (please see individual response shown below for more detailed description)

The cause underlying the lower energy expenditure in NPGPx knockout mice remained to be resolved. One possibility is that the reduced energy expenditure and the lower spontaneous activity are adaptive changes to increased adiposity. Similar phenomenon was also observed in adipose-specific PPARγ knockout mice which displayed marked loss of fat mass and resistance to HFD-induced obesity due to defective adipogenesis. Increased activity and energy expenditure were observed in these mice (reference 8), suggesting an altered adiposity may influence energy expenditure and spontaneous activity. Alternatively, NPGPx deficiency may directly down-regulate energy expenditure through unknown mechanism, leading to the obesity (please also see page 12 last 6 lines to page 13, first 3 lines).

2. Actually quite to the contrary, it can be expected that increased adipogenesis, without an alteration in energy expenditure would improve metabolic health (see glitazone action) as adipocytes would store lipids better and thus reduce circulating lipids.

Answer: The elevated free fatty acid levels were observed in 24-week old knockout mice with long-term excessive fat accumulation. Macrophage infiltration (Fig. R3A) and proinflammatory cytokine genes expression in adipose tissue (Fig. R3B) were also increased in these mice. Chronic inflammation in adipose tissue has been clearly demonstrated to promote lipolysis of adipose tissue (reference 9, 10, 11). Thus, the elevated fatty acid levels observed in 24-week old NPGPx-knockout mice (Fig. 4I) may be secondary to the inflammatory changes of the adipose tissues. The increased fatty acid supply to the liver would lead to enhanced hepatic very-low density lipoprotein production and hence higher circulating triglycerides levels (Fig. 4J). (please also see Supporting Information

Fig. S7A&S7B and page 9, line 4-6 in the revised manuscript)

In addition, we did observed reduced energy expenditure and spontaneous activity in NPGPx knockout mice on HFD, which may contribute, in part, the higher circulating lipids.

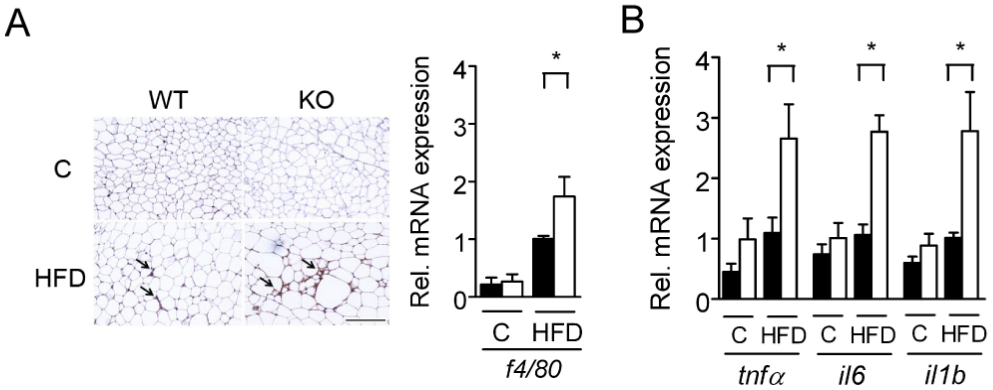

Fig. R3. Macrophage infiltration and pro-inflammatory cytokine gene expression were elevated in NPGPx-knockout mice on HFD. (A) Representative immunohistochemical stain of gonadal fat with anti-F4/80 antibody (arrows, F4/80-positive cells, left panel) and relative expression of *f4/80* mRNA (right panel) in gonadal fat of NPGPx knockout (□) and wild-type mice(■). (B) Relative expression of inflammatory marker genes including *tnfα*, *il6*, and *il1β* mRNA in gonadal fat of NPGPx knockout (□) and wild-type mice (■).

3. The authors here actually report the opposite as adipocyte size is changed while adipocyte number is only marginally increased.

Answer: Indeed, the adipocyte number is marginally increased in NPGPx knockout mice. However, using BrdU labelling of adipose tissues, we found that cellular proliferation is significantly increased in NPGPx-deficient mice as compared to wild-type mice on HFD (as shown in Fig. R7 in response below)

4. Some ideas that could reconcile this problem are the following:

(1) The metabolic cage data is measured after 12 weeks of HFD, maybe a measurement of the mice before HFD reveals a difference that is masked by the HFD effect.

Answer: Energy expenditure was measured at the age of 16 weeks (6 week after HFD). Originally, we found that the daily average energy expenditure was not significantly different between knockout and wild-type mice. We re-analysed the energy expenditure hour-by-hour and found that energy expenditure in the dark/active phase was reduced in knockout mice on HFD as compared to wild-type mice (Fig. R4, right panel). We did not find any difference of energy expenditure between knockout and wild-type mice on chow diet (Fig. R4, left panel) (Supporting Information Fig. S10B and page 9, line 8-10 in the revised manuscript).

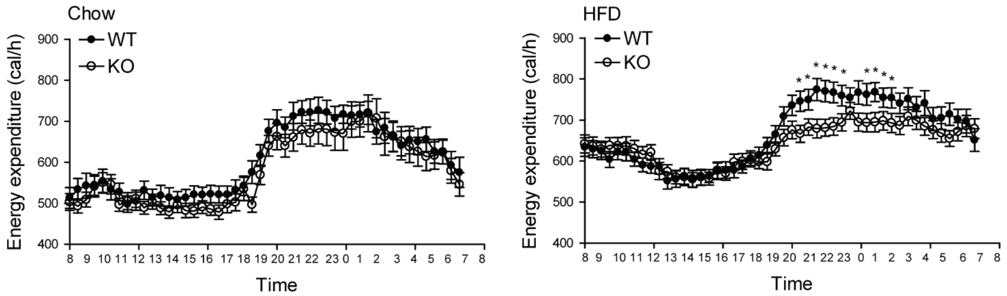

Fig. R4. Energy expenditure of NPGPx-knockout and wild-type mice on chow or high-fat diet (HFD) measured by indirect calorimetry at the age of 16-week (n=15 per group). Values are presented as means ± S.E.M. *P< 0.05. Open bars (□) denote knockout mice and filled bars (■) denote wild-type mice. All values are presented as means ± S.E.M. * P< 0.05

We also noted reduced spontaneous ambulatory activity in NPGPx knockout mice on HFD but not on chow diet (Fig R5A). However, exercise capability measured by rotarod test (Fig. R5B) and

exercise endurance test (Fig. R5C) and was not changed in these mice. In addition, we cannot detect any defect in fatty acid oxidation in primary myoblast (Fig. R5D) and mitochondrial oxidative phosphorylation in primary fibroblasts (Fig. R5E) isolated from NPGPx knockout mice. Furthermore, rectal temperature (Fig. R5F) and the expression level of genes involved in thermogenesis in brown adipose tissue (BAT) were not altered in knockout mice (Fig. R5G). These data indicate that the reduced energy expenditure is not likely due to a primary defect in mitochondrial function or thermogenesis (also see Supporting Information Fig. S9A-9F and page 9, line 10-17 in the revised manuscript).

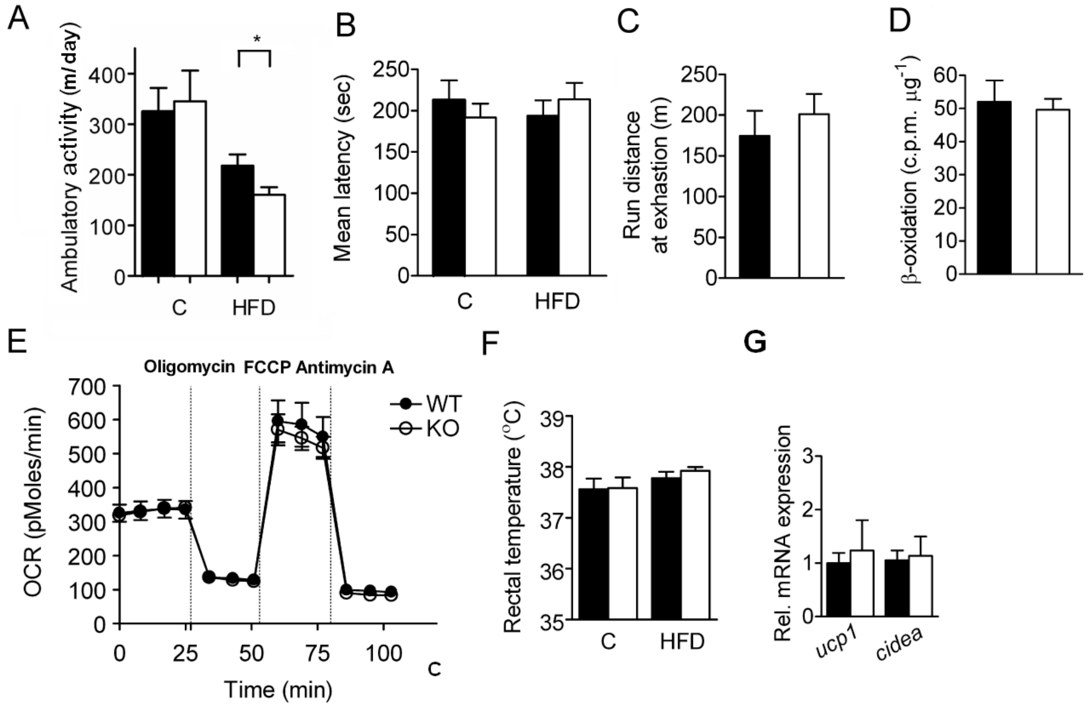

Fig. R5. Ambulatory activity, exercise capability, fatty acid beta-oxidation, mitochondrial oxidative phosphorylation, and thermogenesis genes expression in NPGPx knockout and wild-type mice. (A) Ambulatory activity of 16-week-old mice on chow (C) or high-fat diet (HFD) (*n*=10-15 per group) (B) Running distance at exhaustion of 16-week-old mice in exercise endurance test (*n*=10 per group). (C) Mean latency on the rotarod test of 16-week-old mice (D) Rate of palmitate beta-oxidation in primary myoblasts isolated from knockout mice and wild-type littermates (*n*=10 per group). (E) Oxygen consumption rate (OCR) of mouse embryonic fibroblasts isolated from knockout mice and wild-type littermates (*n*=10 per group). Cells were treated with 2μM oligomycin, 1μM FCCP, and 2μM antimycin A to access mitochondrial oxidative phosphorylation. (F) Rectal temperature of 16-week-old mice (*n*=7-15 per group). (G) Relative expression of genes involved in thermogenesis (*ucp1* and *cidea*) in brown fat of 24-week-old mice on a HFD (*n*=5 per group). Open circles (○) or open bars (□) denote knockout mice. Filled circles (●) or filled bars (■) denote wild-type mice. All values are presented as means ± S.E.M. * $P< 0.05$.

(2) Is the body temperature of the animals changed

Answer: As indicated in response above, body temperature was not changed in NPGPx knockout mice (Fig. R5F or Supporting Information Fig. S9F in the revised manuscript).

(3). Would increased ROS levels in the SVF elicit any kind of response that could lead to alterations in the energy balance, inflammation comes to mind.

Answer: We did not observe any inflammatory response in adipose tissues in NPGPx knockout mice on chow diet. Increased inflammation in adipose tissue of NPGPx-knockout mice was observed after prolonged HFD feeding as compared to wild-type mice (Fig. R3). However, this inflammatory change is less likely to be the cause of the obesity in these mice since most published data suggest that inflammation actually increases energy expenditure and promotes weight loss (reference 12).

(4) Is in vivo adipogenesis affected?
Answer: To address this issue, we injected 5' -bromo-2'-deoxyuridine (BrdU) intra-peritoneally into mice to label proliferating cells. Increased cellular proliferation was observed in gonadal adipose tissue of NPGPx knockout mice (Fig. R6A&B). These data indicate NPGPx deficiency promotes adipogenesis *in vivo*. We have incorporated this data into the main text (also shown in Supporting Information Fig. S6 and page 8, last 2 lines to page 9, line 1).

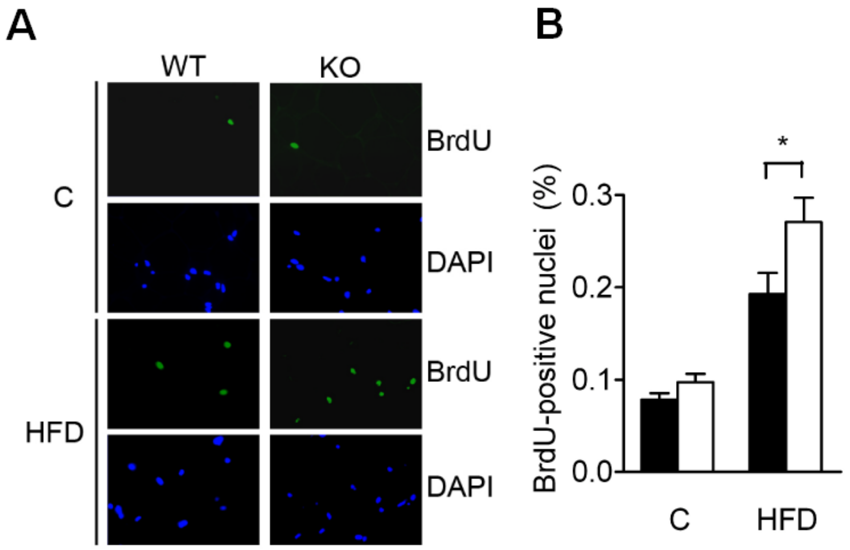

Fig. R6. *In vivo* adipogenesis using 5'-bromo-2'-deoxyuridine (BrdU) labelling (A) Immunofluorescence showing cell proliferation in gonadal fat of wild-type mice (■) and NPGPx knockout mice (□) on chow (C) (*n*=5-9 per group) and high-fat fat (HFD) (*n*=10-15 per group) by BrdU incorporation (green fluorescence). Blue fluorescence indicates DAPI (4',6-diamidino-2-phenylindole) stain. (B) Quantification of the percentage of BrdU-positive nuclei (200 nuclei counted for each mouse).

5. Alternatively, it could well be that another tissue contributes to the observed phenotype which is difficult to prove as there are no usable mouse lines to generate a pre-adipocyte specific knock out mouse.
Answer: As indicated in response above, we did not found defective exercise capability or thermogenesis in NPGPx knockout mice. Mitochondrial respiration and fatty acid oxidation was also not impaired in primary myoblasts and fibroblasts isolated from these mice. However, we still could not exclude the possibility that NPGPx deficiency cause obesity through mechanism other than adipogenesis. This limitation will be fully discussed in the revised manuscript (please see page 12, last 6 lines to page 13, first 3 lines).

Minor points: I would switch Figure 2 with 3 and 4, as this would improve the flow of the manuscript.

Answer: We thank the reviewer for this constructive suggestion and have switched Figure 2 with 3 and 4 (please see the revised manuscript).

Reference

1. Dale R. Nyholt. A simple correction for multiple testing for single-nucleotide polymorphisms in linkage disequilibrium with each other. Am J Hum Genet. 74: 765–769, 2004
2. Gao X, Starmer J, Martin ER. A multiple testing correction method for genetic association studies using correlated single nucleotide polymorphisms. Genet Epidemiol. 32:361-9, 2008
3. Sambrook J, Fritsch E & Maniatis T (2011) Chapter 6. Analysis of Genetic Association Studies. Springer Press, New York, USA.
4. Lettre G, Lange C, Hirschhorn JN. Genetic model testing and statistical power in population-based association studies of quantitative traits. Genet Epidemiol. 31:358-62, 2007.

© EMBO

5. So HC, Sham PC. Robust association tests under different genetic models, allowing for binary or quantitative traits and covariates. Behav Genet. 41: 768–775, 2011
6. Cathryn M. Lewis and Jo Knight (2012) Introduction to Genetic Association Studies. Cold Spring Harb Protoc 2012. Cold Spring Harbor Laboratory Press, New York, USA
7. O'Rahilly S. Human genetics illuminates the paths to metabolic disease. Nature 462: 307-314, 2009
8. Jones JR, Barrick C, Kim KA, Lindner J, Blondeau B, Fujimoto Y, Shiota M, Kesterson RA, Kahn BB, Magnuson MA. Deletion of PPARgamma in adipose tissues of mice protects against high fat diet-induced obesity and insulin resistance. Proc Natl Acad Sci U S A.102:6207-12, 2005
9. Xu H, Barnes GT, Yang Q, Tan G, Yang D, Chou CJ, Sole J, Nichols A, Ross JS, Tartaglia LA, Chen H. Chronic inflammation in fat plays a crucial role in the development of obesity-related insulin resistance. J Clin Invest. 112:1821-1830, 2003
10. Chawla A, Nguyen KD, Goh YP. Macrophage-mediated inflammation in metabolic disease. Nat Rev Immunol. 11:738-749, 2011
11. Suganami T, Nishida J, Ogawa Y. A paracrine loop between adipocytes and macrophages aggravates inflammatory changes: role of free fatty acids and tumor necrosis factor alpha. Arterioscler Thromb Vasc Biol. 25:2062-8, 2005.
12. Ye J, McGuinness OP. Inflammation during obesity is not all bad: evidence from animal and human studies. Am J Physiol Endocrinol Metab. 304:E466-77, 2012

2nd Editorial Decision 29 May 2013

Thank you for the submission of your revised manuscript to EMBO Molecular Medicine. We have now received the enclosed reports from the Reviewers that were asked to re-assess it. As you will see the reviewers are now supportive and I am pleased to inform you that we will be able to accept your manuscript pending the following final amendments:

As per our Author Guidelines, the description of all reported data that includes statistical testing must state the name of the statistical test used to generate error bars and P values, the number (n) of independent experiments underlying each data point (not replicate measures of one sample), and the actual P value for each test (not merely 'significant' or 'P < 0.05').

Please submit your revised manuscript (without the red text in this final version) within two weeks. Needless to say, the sooner we receive it the sooner I will be able to formally accept your manuscript.

I look forward to reading a new revised version of your manuscript as soon as possible.

***** Reviewer's comments *****

Referee #1 (General Remarks):

I have no additional concerns.
The manuscript is very well-written, the design is adequate and i scientifically sound.

Referee #2 (General Remarks):

The authors have done a great job addressing my previous concerns on the SNP association analysis. I don't have additional comments.

Referee #3 (Comments on Novelty/Model System):

The authors addressed all of my concerns in a very thorough manner. I still maintain that the increased adipogenesis is not per se the underlying cause for the observed phenotype, however the novelty especially the role of ROS in adipogenesis is highly important and thus I would support publication of this article.

2nd Revision - authors' response 30 May 2013

Attached please find the revised manuscript (EMM-2013-02679.V3) entitled “Deficiency of NPGPx, an oxidative stress sensor, leads to obesity in mice and human“ by Yi-Cheng Chang et al. We have added the description of all reported statistical data including the name of the statistical test used, the actual P values, and the number (n) of independent experiments (please see the legends for Figure 1E, 1F, 1H, 1I, 1N, 2A, 2F, 3D, 3H, 4A, 4C, 4D, 4F, 4H, 4I, 4J, 4K, 5A, 5B, 5C, 5D, 5E, 5G, 5H, 5I, Supporting Information Figure S3B, S3C, S4A, S5, S6B, S7B, S8B, S9A, S10B, and S11.)

© EMBO
