# Supplementary material for: Deficiency of NPGPx, an oxidative stress sensor, leads to obesity in mice and human
Source: EMBO Mol Med. 2013 Jul 4;5(8):1165–79. doi: 10.1002/emmm.201302679 (PMC3944459; doi:10.1002/emmm.201302679)
Supplement: Supplementary file 2 [file emmm0005-1165-SD2.doc]

**Supporting Information**

**Table of content**

**Supporting Information Fig. S1**

**Supporting Information Fig. S2**

**Supporting Information Fig. S3**

**Supporting Information Fig. S4**

**Supporting Information Fig. S5**

**Supporting Information Fig. S6**

**Supporting Information Fig. S7**

**Supporting Information Fig. S8**

**Supporting Information Fig. S9**

**Supporting Information Fig. S10**

**Supporting Information Fig. S11**

**Supporting Information Table I.**

**Supporting Information Table II.**

**Supporting Information Methods**

**Supporting Information References**

**Supporting Information Fig. S1**

**
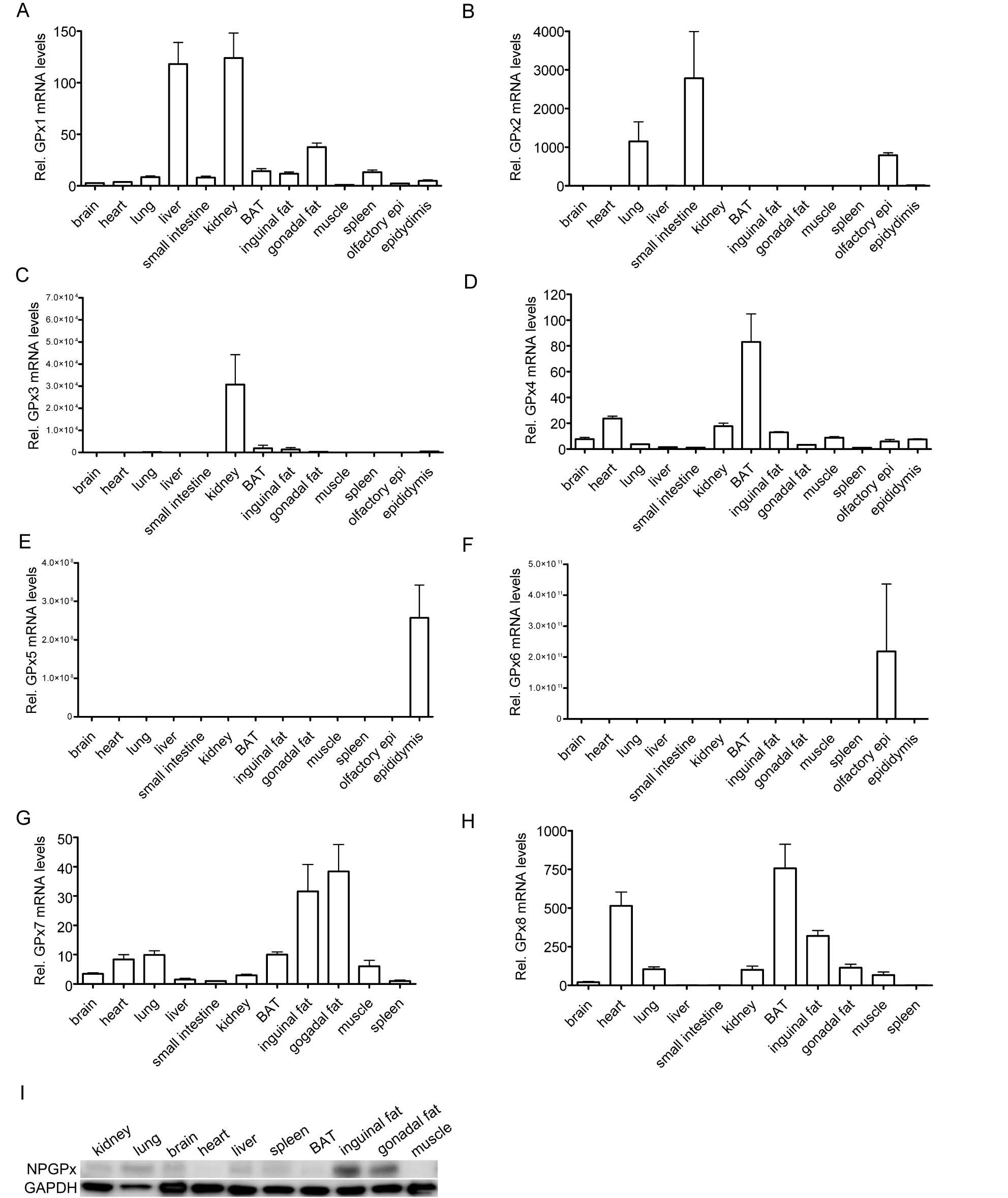
**

**Supporting Information Fig. S1** Relative mRNA levels of (A)GPx1, (B)GPx2, (C)GPx3, (D)GPx4, (E)GPx5, (F)GPx6, (G)GPx7(NPGPx), (H) GPx8 measured by qRT-PCR in various tissues of C57BL/6 mice (*n* =3 per group) (I) GPx7 (NPGPx) tissue expression pattern assayed by Western blots in C57BL/6 mice.

**Supporting Information Fig. S2.**

**
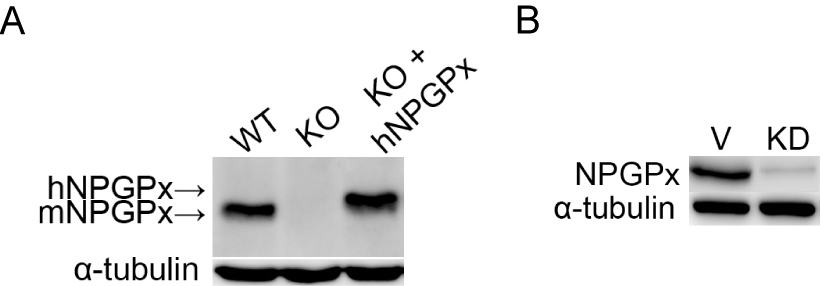
**

**Supporting Information Fig. S2** (A**)** NPGPx expression in wild-type SVF cells(WT), NPGPx-knockout SVF cells (KO), and NPGPx-knockout SVF cells expressing human NPGPx (KO + hNPGPx) (B) NPGPx expression in NPGPx- knockdown (KD) and control (V) 3T3-L1 preadipocytes.

**Supporting Information Fig. S3**

**
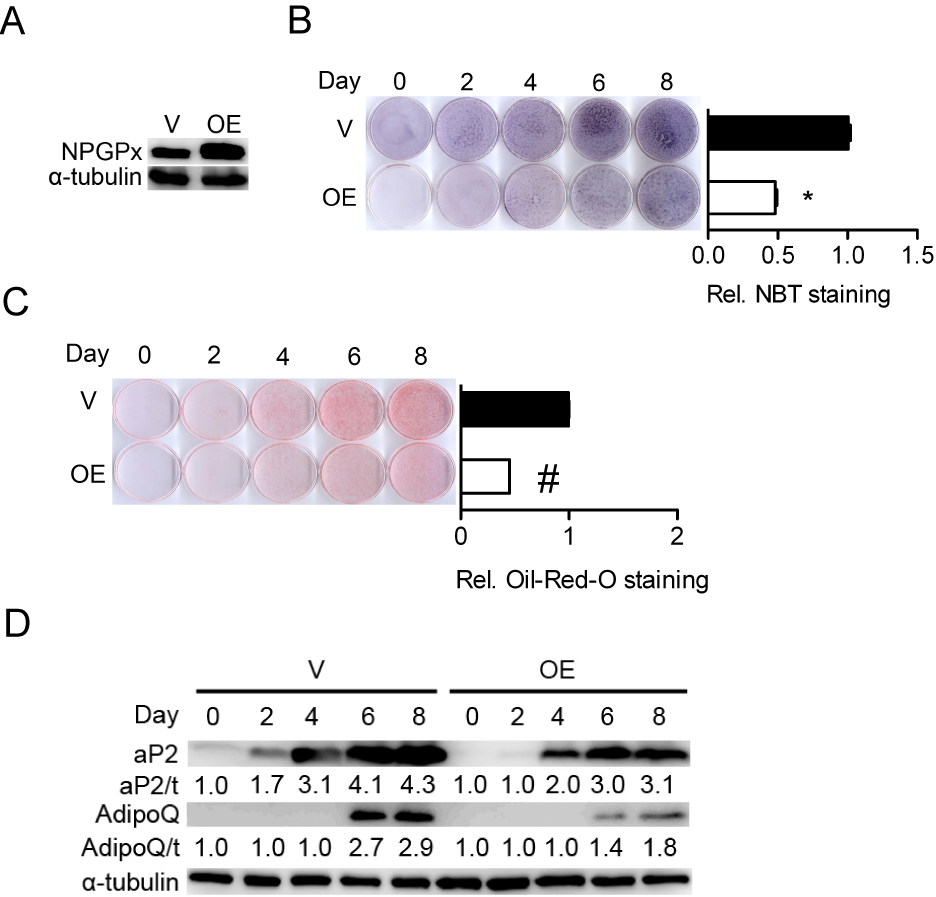
**

**Supporting Information Fig. S3** (A) NPGPx expression in control (V) 3T3-L1 preadipocytes and 3T3-L1 preadipocyte over-expressing NPGPx (OE). (B) NBT reduction stain (C) and Oil Red O staining (right panel) of V and OE 3T3-L1 preadipocytes after induction. Quantification of NBT or Oil-Red O stain at day 8 was shown in bars (*n*=3 per group). **P*< 0.0001 and # *P*=0.008 by independent Student t-tests (D) Western blots showing *ap2* and *adiponectin* expression in V and OE 3T3-L1 preadipocytes after induction. All values are presented as means ± S.E.M..

**Supporting Information Fig. S4**

**
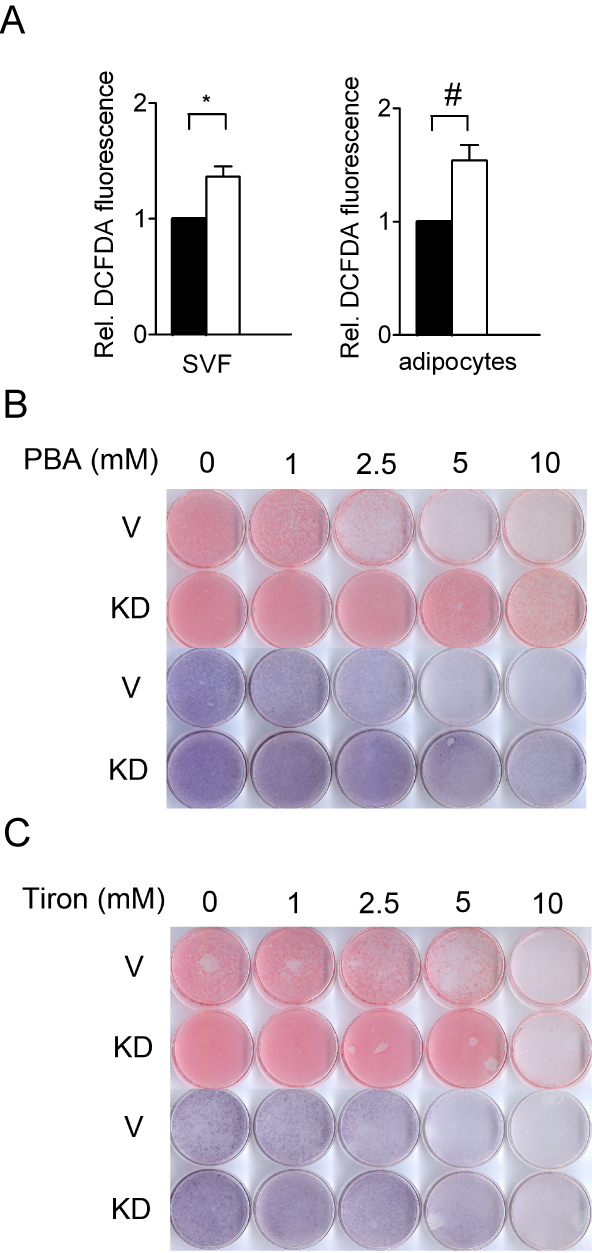
**

**Supporting Information Fig. S4** (A) ROS levels measured by CM-H_2_-DCFDA fluorescence in stromal vascular fraction cells (SVF) and adipocytes isolated from the gonadal fat of wild-type (■) and NPGPx knockout (□) mice (*n*=5 per group). **P*=0.01 and #*P*=0.01 by paired Student t-tests (B&C) Oil Red O staining (upper panels) and nitroblue tetrazolium (NBT) reduction staining (lower panels) of NPGPx-knockdown (KD) and control (V) preadipocytes 8 days after adipogenic induction. (B) 4-phenylbutyrate (PBA) or (C) tiron was added in the first 2 days after induction. All values are presented as means ± S.E.M.

**Supporting Information Fig. S5**

**
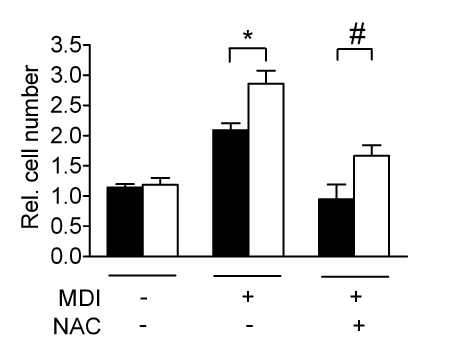
**

**Supporting Information Fig. S5** Cell proliferation measured by bromodeoxyuridine **(BrdU)** incorporation in NPGPx-knockdown (□) and control (■) 3T3-L1 preadipocytes before or 18 hr after adipogenic stimulation (MDI) with or without *N*-acetylcysteine (NAC) treatment. (*n*=3 per group). * *P*=0.02 and # *P*=0.04 by independent Student t-tests

**Supporting Information Fig. S6.**

**
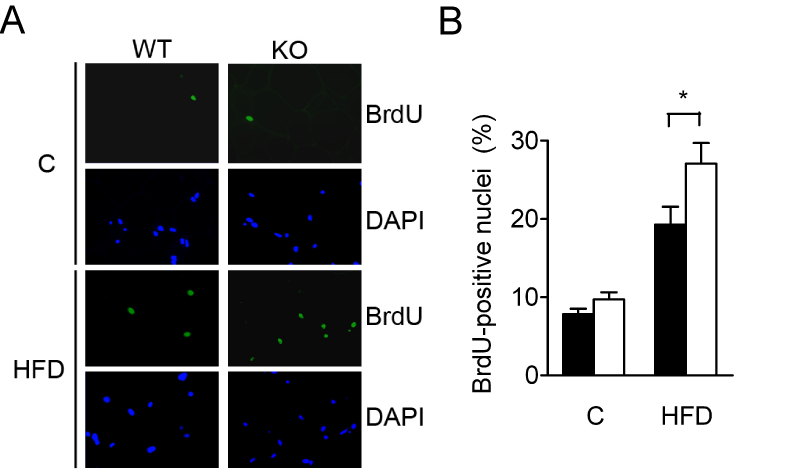
**

**Supporting Information Fig. S6.** (A) Immunofluorescence showing cell proliferation in gonadal fat of wild-type mice (■) and NPGPx knockout mice (□) on chow (C) (*n*=5-9 per group) and high-fat diet (HFD) (*n*=10-15 per group) by BrdU labeling (green fluorescence). Blue fluorescence indicates DAPI (4',6-diamidino-2-phenylindole) stain. (B) Quantification of the percentage of BrdU-positive nuclei (200 DAPI-stained nuclei were counted for each mouse).* *P*=0.03 by independent Student t-test.

**Supporting Information Fig. S7.**

**
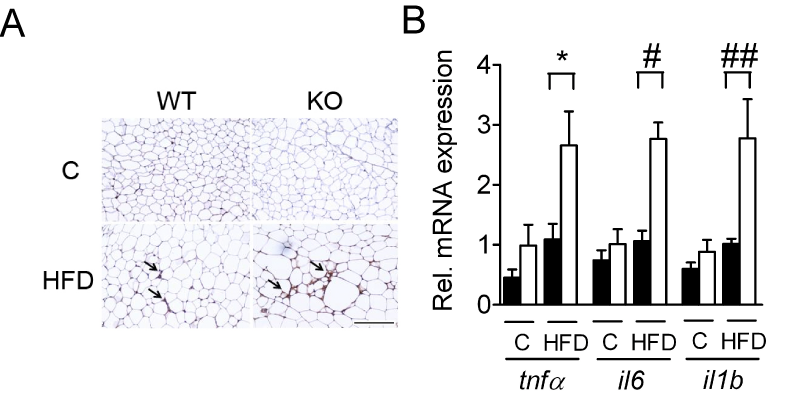
**

**Supporting Information Fig. S7.** (A) Immunohistochemical stain of gonadal fat with anti-F4/80 antibody (arrows, F4/80-positive cells) in gonadal fat of NPGPx knockout (KO) and wild-type mice(WT) on either chow(C) or high-fat diet (HFD) (*n*=5 per group). (B) Relative expression of inflammatory marker genes including *tnfα*, *il6*, and *i*l1β mRNA in gonadal fat of NPGPx knockout and wild-type mice (n=5 per group). **P*=0.03, # *P*=0.001, ## *P*=0.006 by independent Student t-tests. Open bars (□) denote knockout mice and filled bars (■) denote wild-type mice.

**Supporting Information Fig. S8**


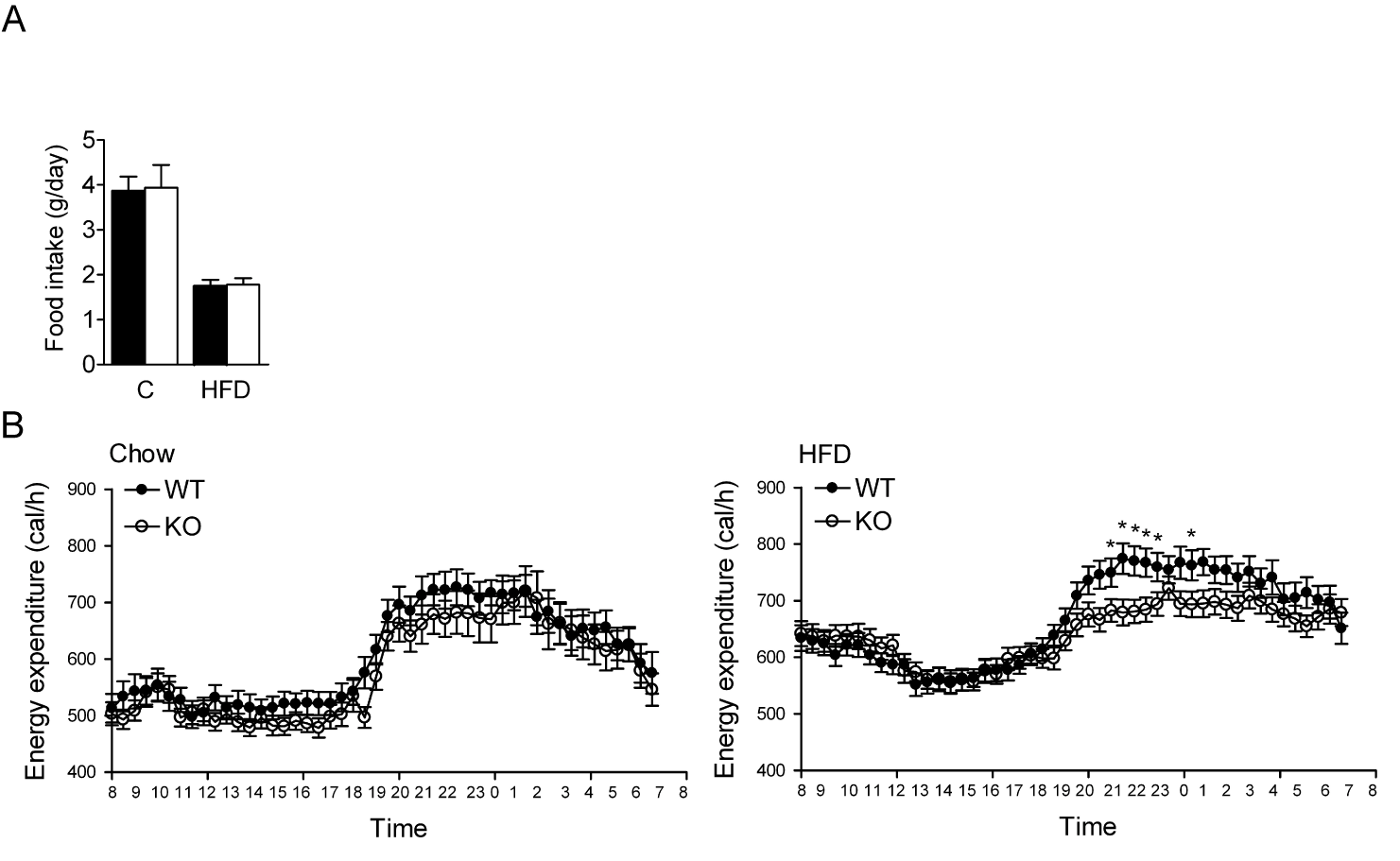


**Supporting Information Fig. S8** (A) Food intake and (B) energy expenditure measured by indirect calorimetry in 16-week-old mice under high-fat diet (HFD) and chow diet (C) (*n*=15 per group). **P*=0.01, 0.01, 0.008, 0.01, 0.04, and 0.03 by independent student t-tests (from left to right). Open bars (□) denote knockout mice and filled bars (■) denote wild-type mice. All values are presented as means ± S.E.M.

**Supporting Information Fig. S9**

**
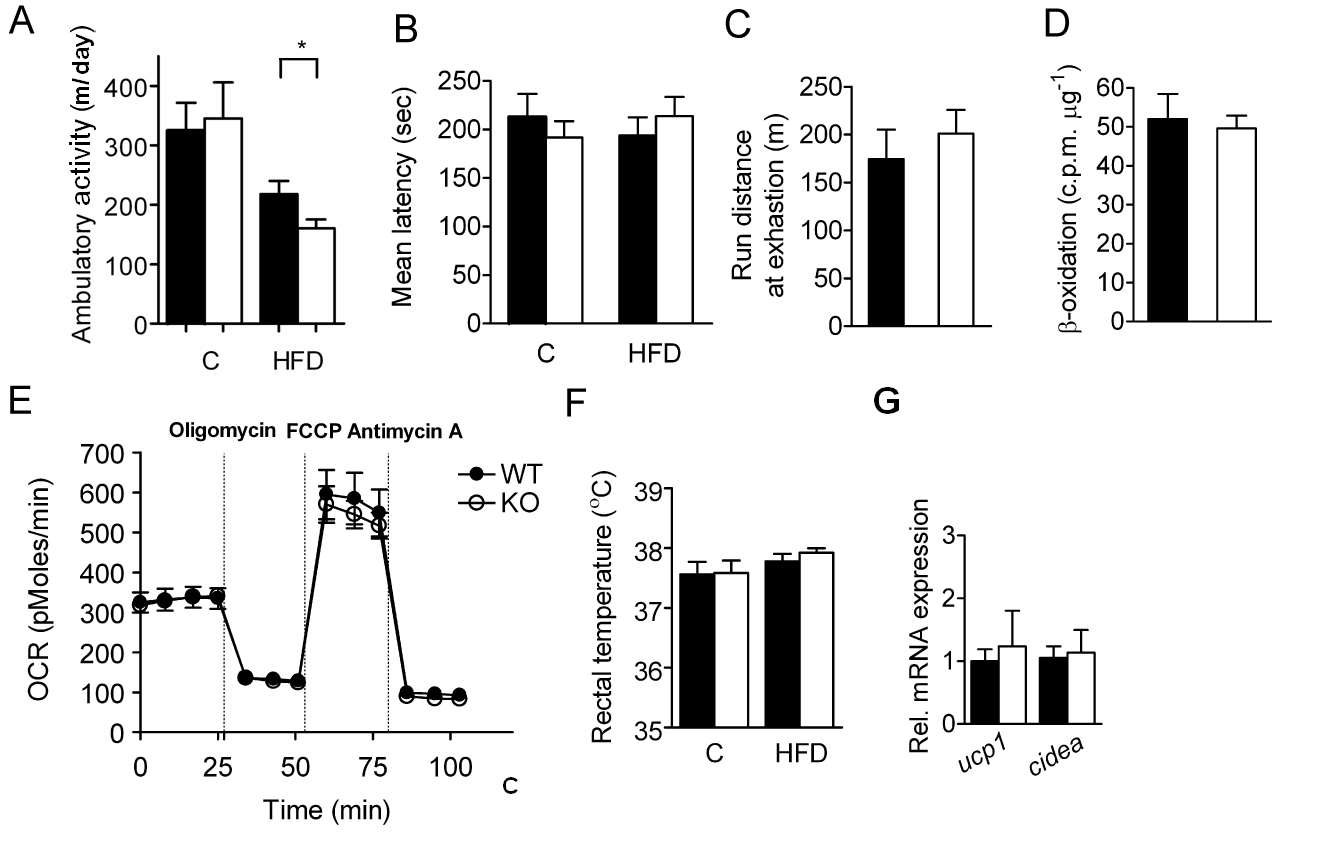
**

**Supporting Information Fig. S9** (A) Ambulatory activity (m/day) of 16-week-old mice on chow (C) or high-fat diet (HFD) (*n*=10-15 per group)**P*=0.03 by independent Student t-test (B) Mean latency on the rotarod test of 16-week-old mice on a chow (C) or high-fat diet (HFD) (*n*=15 per group) (C) Running distance at exhaustion of mice of 16-week-old mice in exercise endurance test on HFD (*n*=10 per group) (D) Rate of palmitate beta-oxidation in primary myoblasts isolated from knockout mice and wild-type littermates (*n*=10 per group). (E) Oxygen consumption rate (OCR) of mouse embryonic fibroblasts isolated from knockout mice and wild-type littermates (*n*=10 per group). Cells were treated with 2μM oligomycin, 1μM FCCP, and 2μM antimycin A to access mitochondrial oxidative phosphorylation. (F) Rectal temperature of 16-week-old mice on a standard chow or a high-fat diet (*n*=7-15 per group). (G) Relative expression of genes involved in thermogenesis (*ucp1* and *cidea*) in brown fat of 24-week-old mice on a HFD (*n*=5 per group). Open circles (○) or open bars (□) denote knockout mice. Filled circles (●) or filled bars (■) denote wild-type mice. All values are presented as means ± S.E.M.

**Supporting Information Fig. S10**

**
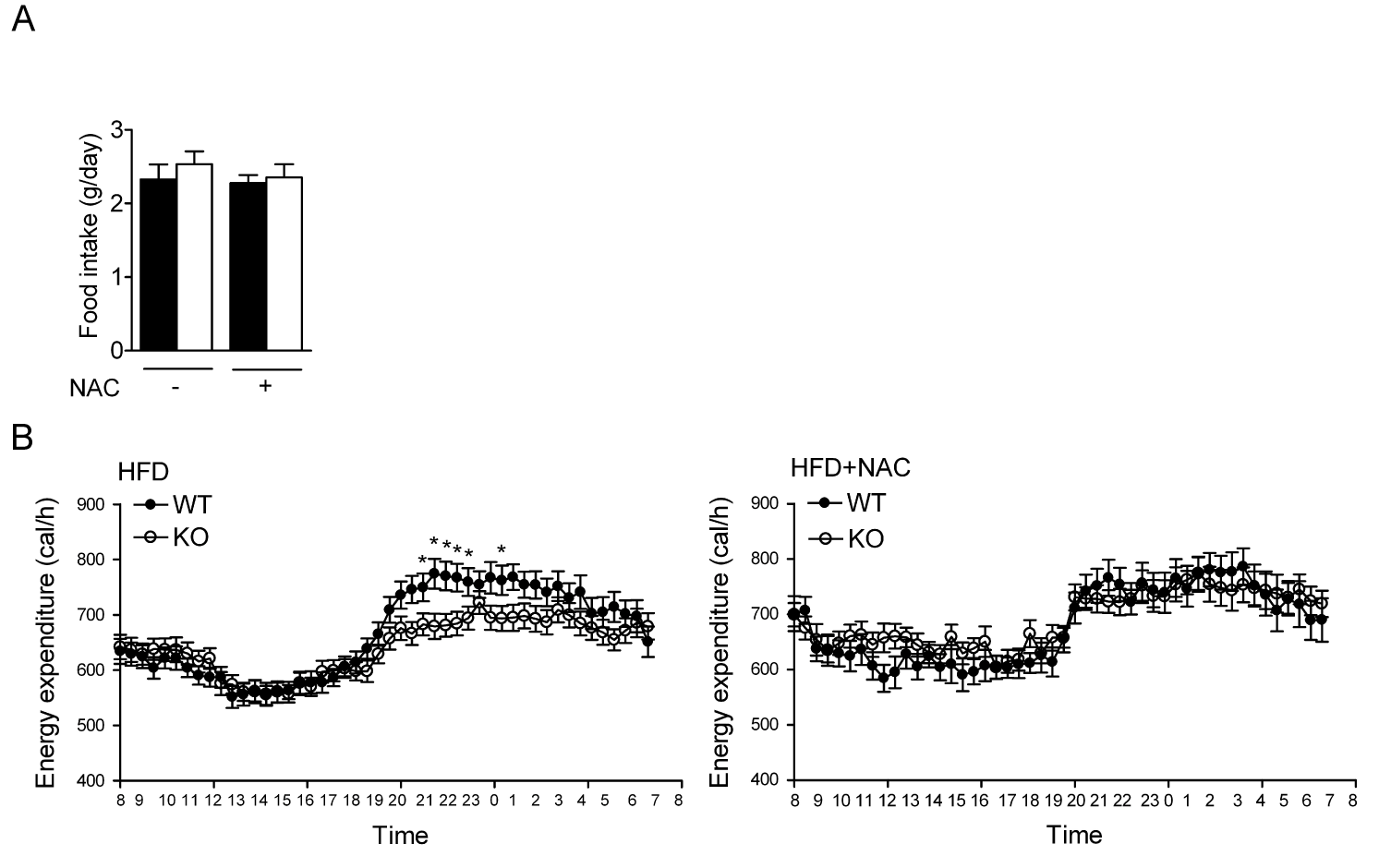
**

**Supporting Information Fig. S10** (A) Food intake (*n*=5-10 per group) and (B) energy expenditure measured by indirect calorimetry of 16-week-old mice (*n*=10 per group). **P*=0.01, 0.01, 0.008, 0.01, 0.04, and 0.03 by independent student t-tests (from left to right).All values are presented as means ± S.E.M. Open bars (□) denote knockout mice and filled bars (■) denote wild-type mice. All values are presented as means ± S.E.M.

**Supporting Information Fig. S11**


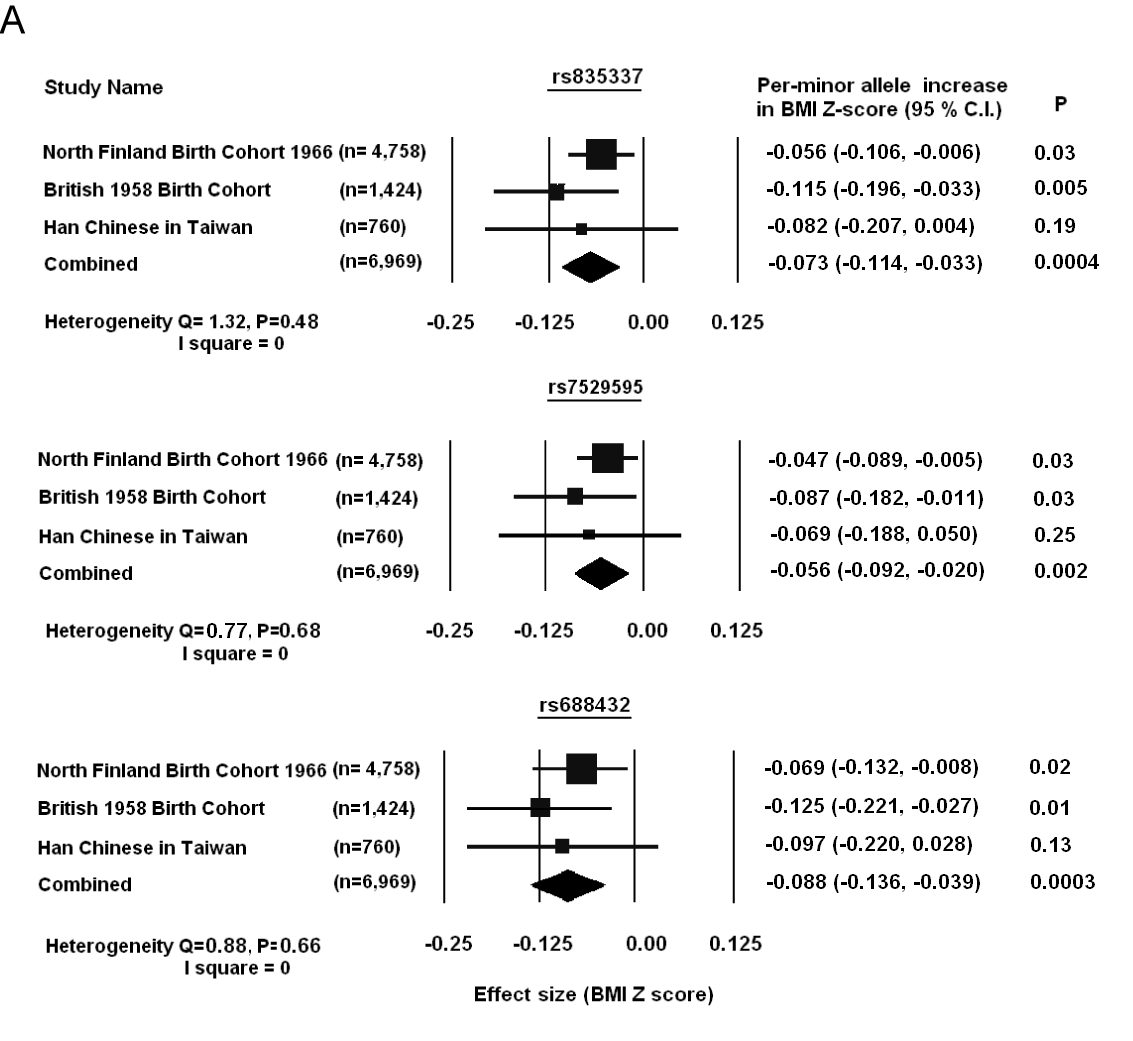


**Supporting Information Fig. S11** Forest plots for meta-analyses of the association between body mass index (BMI) and SNPs near/within the *NPGPx* gene, including (A) rs835337, (B) rs7529595, and (C) rs6588432 in additive models. Estimated per-minor allele effects on BMI are shown in Z score. Meta-analysis was conducted using inverse-variance method for fixed effects. Cochran’s Q and I square were used to measure the heterogeneity between studies.

**Supporting Information Table I.** Body mass index according to rs835337 genotypes

| **Sample population** | **Genotype count (%)** | | | **Body mass index (kg/m^2^)** | | | ***P*-value** |
| --- | --- | --- | --- | --- | --- | --- | --- |
|  | **GG** | **AG** | **AA** | **GG** | **AG** | **AA** |  |
| Han Chinese in Taiwan | 465 (61.3%) | 263 (34.7%) | 30 (3.9%) | 25.00±4.06 | 25.07±3.90 | 23.04±3.16 | 0.006*  (0.008**) |
| Northern Finland Birth Cohort 1966 | 2660(59.5%) | 1570(35.1%) | 240(5.3%) | 24.76±4.22 | 24.59±4.21 | 24.29±4.18 | 0.03 |
| British 1958 Birth Cohort | 740 (52.0%) | 570 (40.0) | 114 (8.0%) | 27.70± 5.04 | 27.26±4.60 | 26.43±4.00 | 0.005 |

All values are presented as means ± S.D. * *P*-value in dominant genetic model ** maximum test (MAX3)-corrected *P*-value

**Supporting Information Table II.** Sequences of primers used in qRT-PCR

| **Gene symbol** | **Gene name** | **Forward (5**′**> 3**′**)** | **Reverse (5**′**> 3**′**)** |
| --- | --- | --- | --- |
| *Gpx1* | Glutathione peroxidase 1 | GTCCACCGTGTATGCCTTCT | CTCCTGGTGTCCGAACTGAT |
| *Gpx2* | Glutathione peroxidase 2 | CCATTGGCCTGGATGGGGAGAAGA | GTTCTGACAGTTCTCCTGATGTCCG |
| *Gpx3* | Glutathione peroxidase 3 | GCCCATCATGCAAGGGCCGA | GGGGTGGGGTAGAATGACTGGGA |
| *Gpx4* | Glutathione peroxidase 4 | AGTACAGGGGTTTCGTGTGC | CGGCAGGTCCTCTATCA |
| *Gpx5* | Glutathione peroxidase 5 | TGGGCTCAAGTATGTTCGTCCAGG | TGAGGGGTGAGGACAAGAACGCT |
| *Gpx6* | Glutathione peroxidase 6 | CGTACCCTGAGCTGAACACATTGCA | TGGGGACATAGCCACCGCCT |
| *Gpx7*  *(Npgpx)* | Glutathione peroxidase 7 | GAACTTTGCCCGCCGCACCT | CAGGTGGGCTCCTTCCCAGAAGT |
| *GPx8* | Glutathione peroxidase 8 | ACGTGGCTAGTGACTGCCGC | ACGCAGGTTCTGCTTCCGGC |
| *18S rRNA* | RNA, 18S ribosomal | ACGATGCCGACTGGCGATGC | TCCTGGTGGTGCCCTTCCGT |
| *Adipoq* | Adiponectin | TCCTGGAGAGAAGGGAGAGAAAG | CCCTTCAGCTCCTGTCATTCC |
| *Fabp4/ap2* | Fatty acid binding protein 4 | GATGCCTTTGTGGGAACCTG | GCCATGCCTGCCACTTTC |
| *Cd36/fat* | Cluster of differentiation 36/ fatty acid translocase | CCCCGTGCCTCCTCCCAGAA | GGCTAGGAAACCATCCACCAGTTGC |
| *Aqp7* | Aquaporin-7 | GGTGATGGCGAAGAGACACA | GCTTCCTGGATGAGGCATTC |
| *Cebpb* | CCAAT/enhancer binding protein, beta | CAAGCTGAGCGACGAGTACA | CAGCTGCTCCACCTTCTTCT |
| *Cebpa* | CCAAT/enhancer binding protein, alpha | GAACAGCAACGAGTACCGGGTA | GCCATGGCCTTGACCAAGGAG |
| *Pparg* | Peroxisome proliferator-activated receptor gamma | CAAGAATACCAAAGTGCGATCAA | GAGCTGGGTCTTTTCAGAATAATAAG |
| *Tnfα* | Tumor necrosis factor | CCTCCCTCTCATCAGTTCTA | ACTTGGTGGTTTGCTACGAC |
| *IL6* | Interleukin-6 | TAGTCCTTCCTACCCCAATTTCC | TTGGTCCTTAGCCACTCCTTC |
| *IL1β* | Interleukin-1β | CTGGAGAGTGTGGATCCCAAG | GGAAGACACGGATTCCATGGTG |

**Supporting Information Methods**

**Immunohistochemical staining**

For immunohistochemical staining (IHC), white adipose tissues was fixed in 4% paraformaldehyde, processed and embedded in paraffin before sectioning. Tissue slices were de-parafinized and treated with Trilogy (Cell Marque # 920P-06) for antigen retrieval. After blocking with PBS with 10% FCS, tissue slices were incubated with primary rat anti-F4/80 antibody (Abcam #ab6640) in 1: 50 dilution at 4°C overnight and then with secondary goat anti-rat antibody (Abcam # ab6844). Commercial IHC Select® HRP/DAB (Merck Millipore # DAB500) kit was used for signal detection.

**BrdU labeling**

Ten-week old wild-type and NPGPx-knockout mice were injected intraperitoneally with BrdU ( 50μg/g) once daily for 10 days. Gonadal fat was harvested and fixed in 4% paraformaldehyde, processed and embedded in paraffin before sectioning. Tissue slices were de-parafinized, treated with Trilogy (Cell Marque # 920P-06) for antigen retrieval, and then incubated with PBS with 0.2% Triton X100. After blocking with PBS with 10% FCS, tissue slices were incubated with primary rat anti-BrdU antibody (GeneTex #GTX26326) in 1: 200 dilution at 4°C overnight and then with secondary rabbit Alexa Fluor® 488 anti-rat antibody (Molecular Probe # A-11006). Tissue slices were then incubated with DAPI (4',6-diamidino-2-phenylindole) with 1: 5000 dilution of stock solution for 10 min. Immunofluorescent signals were acquired using excitation 488 nm/emission 519 nm for BrdU staining and excitation 359 nm/ emission 461 nm for DAPI staining. The percentages of BrdU-positive nuclei in 200 consecutive DAPI-stained nuclei were counted for each fat tissue.

**Mouse behavior analysis**

The behaviors of mice were monitored using a digital video monitoring system. Four CCD cameras (Focus SNO39A, S/N:R720166) were mounted perpendicular to the cages. The signal from cameras were transferred to a recording system, 4-CH-DVMR (Model: TA-462), connected to the WinTV-PVR recording computer. During the recording period, mice were housed in standard cages, with minimal bedding (100 ml) to minimize mounding. Mice were recorded for 24 hrs after acclimation for more than 16 hrs. Video data were analyzed by HomeCageScan software (Clever Systems, Reston, VA). The automated analysis built a profile of animal behaviors, including ambulatory activity, hanging, rearing, drinking, and feeding for each mouse.

**Exercise endurance test**

Animals were acclimatized to the test using a habituation protocol the day preceding the running test. Mice were undisturbed for 15 minutes. Mice were then trained and warmed up on a treadmill at 1-2 m/min for 5 minutes, followed by acceleration of 1 m/min to a final speed of 24 m/min. Mice ran at this speed until exhausted. The distance run and the number of shocks obtained were recorded. A mouse was considered exhausted and removed from the experiment when it stayed on the shock grid without attempting to re-engage the treadmill for more than 5 sec.

**Palmitate beta-oxidation assay**

Mouse myoblasts were prepared from the quardriceps muscle of postnatal mice and was induced to differentiate into myofibrils. For the palmitate beta-oxidation assay, myoblasts were incubated in phosphate buffered saline (PBS) (with Ca^2+^ and Mg^2+^) containing 125 μM palmitic acid (Sigma #9767), 1% fatty-acid-free bovine serum albumin (BSA) (Sigma #A6003), 1 mM carnitine (Sigma #C0283) and 0.002 mCi/mL of [9,10-^3^H]-palmitic acid (Perkin Elmer #NET-043) at 37°C for 2 hrs. At the end of incubation, 200 μL of supernatant was transferred into a centrifugation tube and 0.75 mL chloroform/methanol (1:2) was added followed by 0.25 mL chloroform and 0.25 mL of 2 M KCl/ HCl solution. After centrifugation, the upper aqueous layer was treated with a mixture of 0.4 mL chloroform, 0.4 mL methanol, and 0.36 mL of 2 M KCl/HCl. After centrifugation, the upper aqueous layer containing ^3^H_2_O was transferred to a scintillation vial. Total ^3^H_2_O amount was determined by β counter. The cell was lysed for protein quantification according to the Bradford assay. The palmitate oxidation rate was calculated as β count normalized by the total protein content.

**Measurement of mitochondrial oxidative phosphorylation**

Mouse embryonic fibroblasts (MEF) were prepared from 13.5d embryos. Embryos were chopped and digested in trypsin at 37°C for 30 minutes. The digested cells were washed and cultured following the NIH 3T3 culture protocol in which 10^6^ cells were passed into a 10-cm dish every 3 days. Rate of oxidative phosphorylation were determined by measuring oxygen consumption rate (OCR) using an XF24 XF analyzer (Seahorse Bioscience) according to manufacturer’s instruction. Briefly, 3 × 10^5^ MEF/well were plated in XF 24-well cell culture microplates (Seahorse Biosciences #100777-004) and incubated at 37 °C overnight. One hour before OCR analysis, medium was changed to XF Assay Medium (Seahorse Biosciences # 102352-000) supplemented with 5% FBS and glucose (4.5g/L). Oligomycin, FCCP, and antimycin A were loaded into drug delivery ports and added sequentially at the indicated time points.

**Isolation of preadipocytes, macrophages, and endothelial cells**

Mouse white adipose tissue was harvested and digested with type I collagenase at 37°C in a shaking water bath for 60 min. SVF and adipocytes fraction were separated by centrifugation. CD11b^+^ (macrophage marker) cells and CD31^+^ (endothelial cell marker) cells were purified separately from the SVF by positive selection using biotin-conjugated CD11b anti-mouse antibody (Invitrogen #RM2815), biotin-conjugated anti-mouse CD31 antibody (eBioscience # 13-0311-85), and the Dynabeads® Biotin Binder kit (Invitrogen, # 11047). Preadipocytes (Lin^-^CD34^+^CD29^+^Sca1^+^) were obtained from the SVF by depletion of lineage- positive (Lin+: CD31^+^, CD45^+^, and Ter119^+^) population using biotin conjugated antibody Ter119 and CD45 (eBioscience #13-5921-82, and #13-0451-82) and then further purified for CD34^+^, CD29^+^ and Sca-1^+^ positive cells using biotin conjugated antibodies against CD34, CD29, and Sca-1 (eBioscience #13-0341-82, # 13-0291-82, #13-5981-82) and the CELLection™ Biotin Binder kit (Invitrogen # 11533D).

**Supporting Information References**

Rodeheffer MS, Birsoy K, Friedman JM (2008) Identification of white adipocyte progenitor cells in vivo. Cell 1**3**5: 240-249
